# Synthesis of the spiroketal core of integramycin

**DOI:** 10.3762/bjoc.9.282

**Published:** 2013-11-12

**Authors:** Evgeny V Prusov

**Affiliations:** 1 Department of Medicinal Chemistry, Helmholtz Centre for Infection Research, Inhoffenstr. 7, 38124 Braunschweig, Germany

**Keywords:** anti-infectives, hydrozirconation, natural products, spiroketals, total synthesis

## Abstract

A concise synthetic strategy towards the spiroketal core of the HIV-integrase inhibitor integramycin (**1**) was developed. The required ketone precursor was efficiently constructed from two simple and easily accessible subunits by means of a hydrozirconation/copper catalyzed acylation reaction. The effects of different protecting groups on the spiroketalization step were also investigated.

## Findings

**Figure 1:**
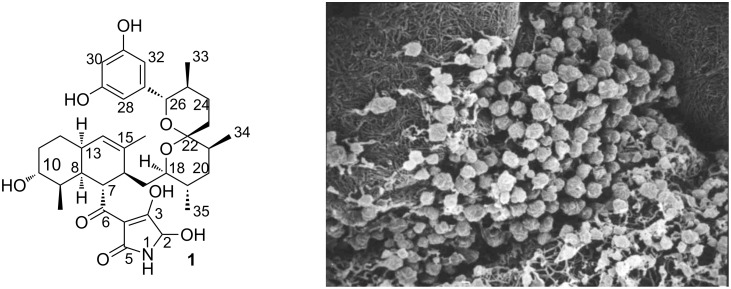
Structure of integramycin (**1**) and its producing organism, *Actinoplanes sp.* (*Photo: J. Wink, HZI, Microbial drugs*).

Integramycin is a polyketide natural product isolated from *Actinoplanes sp*. by the Singh group at Merck [1] (Figure 1). The compound was found to inhibit HIV-1 integrase coupled strand transfer reactions with IC_50_ values of 3 and 4 μM, respectively. Structurally, the molecule architecture of integramycin features four distinct elements: a 3,5-dihydroxy-substituted aromatic ring, a spiroketal core, a highly functionalized octalin moiety and a 5-hydroxylated 3-acyltetramic acid. Studies on the syntheses of the spiroketal core [2–3] and the octalin fragment [4] of integramycin were recently reported by Roush and Floreancig, however, a total synthesis of this rather complex natural product has yet to be accomplished. Intrigued by its biological properties and capitalizing on our previous experience [5] in the total synthesis of natural products with tetramic acid fragments we embarked on the development of a concise and modular approach towards integramycin and related natural products.

**Figure 2:**
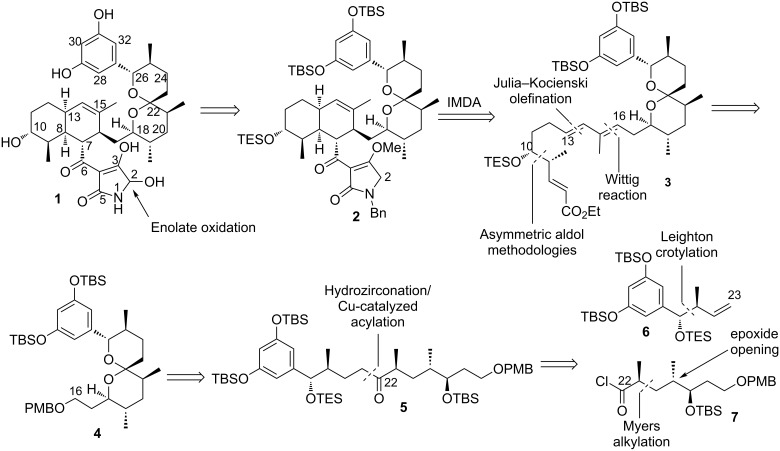
Retrosynthetic analysis of integramycin.

According to the retrosynthetic analysis, which is outlined in Figure 2, the target molecule may be assembled from an appropriate spiroketal advanced intermediate **4.** This spiroketal compound is logically traced back to ketone **5**, which can be further divided in the vicinity of the C22 carbonyl group into two readily accessible building blocks **6** and **7**. For the construction of the octalin fragment we planned to use two sequential olefination reactions followed by an intramolecular Diels–Alder cycloaddition. The installation of the tetramic acid moiety would proceed via Lassey–Dickmann condensation with subsequent introduction of the 5-hydroxy group by oxidation of the corresponding vinylogous enolate. Herein a straightforward synthesis of the C16–C35 spiroketal core of integramycin based on a hydrozirconation/acylation reaction sequence is reported.

**Scheme 1:**
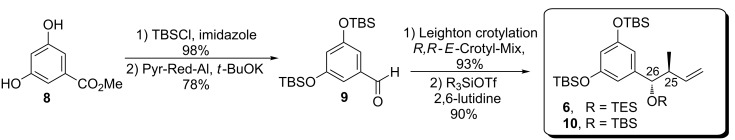
Synthesis of the aromatic subunit.

The synthesis of the aryl-substituted part of the spiroketal fragment was started from 3,5-dihydroxybenzoate **8** which was converted into aldehyde **9** via protection and partial reduction [6] (Scheme 1). Subsequent Leighton crotylation [7] using commercially available (*R*,*R*)-*E*-Crotyl-Mix was employed to introduce the two stereocenters at C25/C26 with *anti*-relationship in excellent yield and enatioselectivity. The enantiomeric excess of the crotylation product was estimated by esterification with (*R*)-α-methoxyphenylacetic acid with subsequent NMR analysis and found to be close to 90%. The resulting alcohol was protected as TBS and TES ethers to investigate the cleavage regime of different protecting groups during the acid catalyzed ketalization step.

**Scheme 2:**
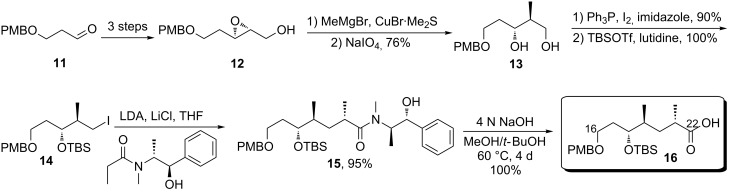
Sharpless epoxidation/Myers alkylation approach to the C16–C22 carboxylic acid fragment.

The second part of the spiroketal core was constructed from the PMB-protected 3-hydroxypropanal via Horner–Wadsworth-Emmons olefination, reduction to the allylic alcohol and Sharples epoxidation [8] (Scheme 2). Subsequent Cu-catalyzed epoxide-opening using methylmagnesium bromide [9] produced an inseparable mixture of 1,2- and 1,3-diol products, which upon treatment with sodium periodate furnished pure **13**. The primary hydroxy group was selectively converted to alkyl iodide by using Ph_3_P/I_2_/imidazole conditions [10]. Then the protected iodide **14** was subjected to Myers alkylation [11] reaction with an excess of enolate to install the last required stereocenter. Hydrolytic cleavage of the pseudoephedrine auxiliary by prolonged exposure to sodium hydroxide at elevated temperature afforded the carboxylic acid **16**.

**Scheme 3:**
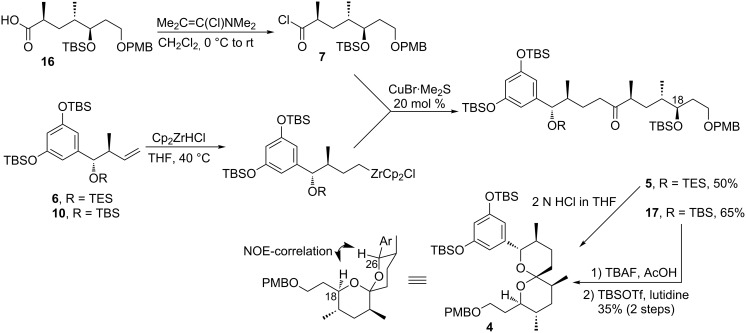
Coupling of the fragments and spiroketalization.

With both fragments in hand, the key coupling was attempted. Initially, the conditions recently reported by Vasse [12] were investigated (Scheme 3). In the event, treatment of the carboxylic acid **16** with Ghosez reagent [13] provided the corresponding acid chloride **7**, which was allowed to react with the hydrozirconation products of alkenes **6** and **10** in dichloromethane in the presence of a stoichiometric amount of CuBr·Me_2_S. The reaction was rather capricious and produced only modest amounts of the target compound. However, application of the original Wipf protocol [14] resulted in a much more favourable outcome and, upon some further optimization of the catalyst load, provided the desired ketone in 65% yield for the TBS protected alkene. The yield for the TES-protected alkene was somewhat lower, probably due to a partial cleavage of the protecting group during the acylation or hydrozirconation step.

Finally, several conditions were studied for the spiroacetalization step. Exposure of the *all*-TBS protected ketone **17** to moderately strong acids, e.g. the HF–pyridine complex in THF or CSA in THF/H_2_O mixture, resulted in the predominant cleavage of the TBS group at C18, whereas the benzylic TBS ether remained mostly untouched. Application of more acidic conditions (3 N HCl in THF) led to a messy reaction. However, treatment of the same ketone with an excess of TBAF buffered with acetic acid caused removal of all protecting groups with concomitant spiroketalization and upon reinstallation of the phenolic protecting groups the desired compound **4** was obtained in 35% yield. The stereochemistry of the spiroketal was confirmed by observation of the strong NOE-correlation for H-18 and H-22 protons in the NOESY spectrum of **4** (see Supporting Information File 1). In the case of the ketone with the TES-protected benzylic alcohol, exposure to 2 N HCl in THF resulted in formation of numerous products, including the spiroketal **4**, thus demanding further optimization of this reaction.

In summary, an efficient route to the C16–C35 spiroketal fragment of integramycin was developed. The key step of the synthesis is a Cu-catalysed coupling of an acid chloride with an alkyl zirconium species. The stereocenters were introduced using Leighton crotylation, Sharpless epoxidation and Myers alkylation. Although the presented route achieves approximately the same overall yield of target compound as that of Floreancig [2] (11.2 vs 11.5%, respectively), it features utilization of easily scalable transformation and upon further optimization of the spiroketalization step would be advantageous for the preparation of intermediate **4**. Efforts towards the completion of the total synthesis of integramycin are currently underway in our laboratory and will be reported in due course.

## Supporting Information

File 1 Experimental procedures.

File 2 Copies of NMR spectra.
