# Supplementary material for: Synthesis of the spiroketal core of integramycin
Source: Beilstein J Org Chem. 2013 Nov 12;9:2446–50. doi: 10.3762/bjoc.9.282 (PMC3869345; doi:10.3762/bjoc.9.282)
Supplement: File 1 — Experimental procedures. [file Beilstein_J_Org_Chem-09-2446-s001.pdf]

Supporting Information

for

Synthesis of the spiroketal core of integramycin

Evgeny. V. Prusov*

Address: Department of Medicinal Chemistry, Helmholtz Centre for Infection Research, Inhoffenstr. 7, 38124 Braunschweig, Germany

Email: Evgeny. V. Prusov - evgeny.prusov@helmholtz-hzi.de

*Corresponding author

Experimental procedures

General remarks

Optical rotations were determined on a Perkin-Elmer 241 instrument. NMR spectra were recorded in $CDCl_3$ on a Bruker AM 300, AM 400, AMX 500, DMX-600 and AMX 700 spectrometer. ESI mass spectra were obtained on a Finnigan MAT 95 spectrometer, high resolution data were acquired using peak matching (M/DM = 10000). Analytical TLC (TLC aluminium sheets silica gel Si 60 F254 (Merck), solvent: mixtures of ethyl acetate/petroleum ether, detection: UV absorption at 254 nm, dark blue spots on staining with cerium(IV)sulfate-phosphomolybdic acid in sulfuric acid followed by charring. Unless otherwise stated, all reactions were performed under inert gas blanket. All solvents used were commercial absolute solvents over molecular sieves with water content less than 50 ppm (e.g. from Acros Organics).

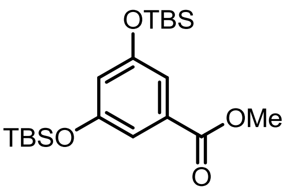

Methyl 3,5-bis(*tert*-butyldimetylsilyloxy)benzoate.

To a stirred solution of methyl 3,5-dihydroxybenzoate (**8**, 9.1 g, 55 mmol) and imidazole (14 g, 220 mmol) in CH_2Cl_2 (100 mL) was added portion-wise *tert*-butyldimethylsilyl chloride (TBSCl, 16.5 g, 110 mmol) and the resulted mixture was stirred at rt overnight. The reaction

was quenched with water (100 mL), the organic phase was separated, and the water phase extracted with CH_2Cl_2 (30 mL). The combined organic layers were washed with water (50 mL), sat. aq. $NaHCO_3$ (25 mL), brine (25 mL), and dried over anhydrous sodium sulfate. The solvent was removed in vacuo, and the crude product was purified by silica-gel column chromatography using hexane/ethyl acetate 97:3 as an eluent to afford methyl 3,5-bis(*tert*-butyldimetylsilyloxy)benzoate (20 g, 98%).

^{1}H NMR (300 MHz, $CDCl_3$) δ = 7.11 (d, *J*=2.3 Hz, 2H), 6.51 (t, *J*=2.3 Hz, 1H), 3.87 (s, 3H), 0.97 (s, 18H), 0.19 (s, 12H)

^{13}C NMR (75MHz, $CDCl_3$) δ = 166.9, 156.6, 131.8, 116.9, 114.6, 52.2, 25.7, 18.3, -4.4.

HRMS: (ESI+) calc. for $C_{20}H_{36}O_4Si_2Na$: 419,2044, found: 419,2042.

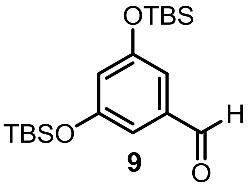

3,5-Bis(*tert*-butyldimetylsilyloxy)benzaldehyde (**9**)

A solution of pyrrolidine (3.5 g, 4 mL, 50 mmol) in MTBE (25 mL) was added dropwise to a 66% toluene solution of Red-Al (14.5 mL, 50 mmol) in MTBE (50 mL) around −20 °C over 20 min and stirred at room temperature for 1 h. A solution of potassium *tert*-butoxide (565 mg, 5 mmol) in THF (5 mL) was added to the mixture. The above reducing agent was added dropwise to a solution of methyl 3,5-bis(*tert*-butyldimetylsilyloxy)benzoate (10 g, 25 mmol) in MTBE (15 mL) around 10 °C over 1 h and stirred for 2 h. The reaction mixture was quenched by pouring into stirred mixture of ice, Et_2O (100 ml) and 2 N HCl (150 mL) and the organic layer was separated. The organic phase was washed with 1 N HCl (50 mL), sat. aq. $NaHCO_3$ (25 mL), brine (25 mL), and dried over anhydrous sodium sulfate. The solvents were evaporated and the residue purified by column chromatography on SiO_2 using hexane/ethyl acetate 97:3 as an eluent to give 3,5-bis(*tert*-butyldimetylsilyloxy)benzaldehyde (7.2 g, 78%) as colorless oil.

^{1}H NMR (400 MHz, $CDCl_3$) δ = 9.87 (s, 1H), 6.96 (d, *J*=2.0 Hz, 2H), 6.60 (t, *J*=2.3 Hz, 1H), 1.00 (s, 18H), 0.22 (s, 12H).

^{13}C NMR (101 MHz, $CDCl_3$) δ = 191.9, 157.3, 138.4, 118.5, 114.4, 25.7, 18.2, -4.4.

HRMS: (ESI+) calc. for $C_{19}H_{35}O_3Si_2$: 367.2125, found: 367.2123.

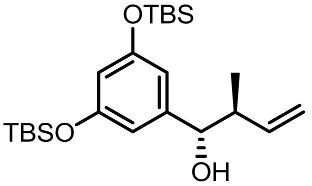

(1*S*,2*S*)-1-(3,5-bis(tert-butyldimethylsilyloxy)phenyl)-2-methylbut-3-en-1-ol

To a solution of (*R*,*R*)-*trans* EZ-CrotylMix (*Sigma-Aldrich* cat. Nr. 737674, 5 g, 7.7 mmol) in CH_2Cl_2 (80 mL) was added 3,5-bis(*tert*-butyldimetylsilyloxy)benzaldehyde (2.82 g, 7.7 mmol). The mixture was stirred vigorously for 30 min and then concentrated. The residue was treated with Et_2O and 1 M HCl, and the resulting mixture was stirred vigorously for 1 h. The mixture was filtered (with Et_2O washes) to recover the diamine as its bis HCl salt (in 95% yield) and the layers of the biphasic filtrate were separated. The aqueous phase was extracted with Et_2O, and the combined organic phases were dried over anhydrous sodium sulfate, filtered, and concentrated. Purification of the crude product by column chromatography with SiO_2 using PE/ethyl acetate 12:1 as an eluent afforded pure alcohol (3.0 g, 93%) as colorless oil.

^{1}H NMR (600 MHz, $CDCl_3$) δ = 6.43 (d, *J*=2.2 Hz, 2H), 6.25 (t, *J*=2.2 Hz, 1H), 5.83 - 5.73 (m, 1H), 5.18 - 5.12 (m, 2H), 4.24 (d, *J*=7.7 Hz, 1H), 0.97 (s, 18H), 0.87 (d, *J*=7.0 Hz, 3H), 0.18 (s, 12H)

^{13}C NMR (101 MHz, $CDCl_3$) δ = 156.4, 144.7, 140.7, 116.6, 112.0, 111.4, 77.7, 46.2, 25.7, 18.3, 16.5, -4.4.

$[\alpha]_D$ = -29.7 (c 1.5, CH_2Cl_2)

HRMS: (ESI+) calc. for $C_{23}H_{43}O_3Si_2$: 423.2749, found: 423.2751.

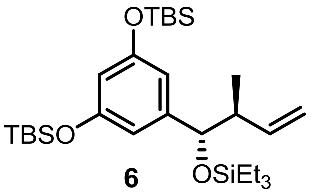

6

(5-((1*S*,2*S*)-2-methyl-1-(triethylsilyloxy)but-3-enyl)-1,3-phenylene)bis(oxy)bis(tert-butyldimethylsilane) (**6)**

To a stirred solution of foregoing alcohol (3 g, 7.2 mmol) and 2,6-lutidine (1.7 mL, 15 mmol mmol) in CH_2Cl_2 (25 mL) at 0 °C was added dropwise triethylsilyl triflate (TESOTf, 2.3 mL, 10 mmol) and the resulted mixture was stirred at 0 °C for 1 h. The reaction was quenched with water (25 mL), the organic phase was separated, and the water phase extracted with CH_2Cl_2 (3 mL). Combined organic layers were washed with 1 N HCl (2 × 20 mL), sat. aq. $NaHCO_3$ (25 mL), brine (25 mL), and dried over anhydrous sodium sulfate. The solvent was removed in vacuo, and the crude product was purified by silica-gel column chromatography using petroleum ether/ethyl acetate 25:1 as an eluent to afford alkene **6** (3.5 g, 90%) as colorless oil.

^{1}H NMR (500 MHz, $CDCl_3$) δ = 6.38 (d, *J*=2.1 Hz, 2H), 6.21 (t, *J*=2.3 Hz, 1H), 5.83 (ddd, *J*=7.4, 10.3, 17.4 Hz, 1H), 4.97 - 4.90 (m, 2H), 4.29 (d, *J*=6.3 Hz, 1H), 2.44 - 2.28 (m, 1H), 0.96 (s, 18H), 0.89 - 0.81 (m, 12H), 0.54 - 0.45 (m, 6H), 0.16 (s, 12H).

^{13}C NMR (150 MHz, $CDCl_3$) δ = 155.9, 146.1, 141.3, 114.2, 112.4, 111.2, 78.9, 46.1, 25.8, 18.3, 15.9, 6.9, 4.9, -4.3, -4.3.

$[\alpha]_D$ = -27.0 (c 3.4, CH_2Cl_2)

HRMS: (ESI+) calc. for $C_{29}H_{57}O_3Si_3$: 537.3612, found: 537.3615.

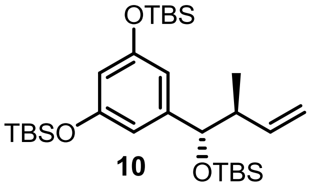

10

(5-((1*S*,2*S*)-1-(*tert*-butyldimethylsilyloxy)-2-methylbut-3-enyl)-1,3-phenylene)bis(oxy)bis(tert-butyldimethylsilane) (**10**)

Compound **10** was obtained in 90% yield according to the previous procedure using *tert*-butyldimethylsilyl triflate instead of triethylsilyl triflate.

^{1}H NMR (600 MHz, $CDCl_3$) δ = 6.37 (d, *J*=2.2 Hz, 2H), 6.21 (t, *J*=2.2 Hz, 1H), 5.81 (ddd, *J*=7.7, 10.1, 17.4 Hz, 1H), 4.95 (d, *J*=10.6 Hz, 1H), 4.91 (d, *J*=17.2 Hz, 1H), 4.28 (d, *J*=6.2 Hz, 1H), 2.39 - 2.31 (m, 1H), 0.96 (s, 18H), 0.87 (s, 9H), 0.85 (d, *J*=7.0 Hz, 3H), 0.16 (s, 12H), 0.01 (s, 3H), -0.19 (s, 3H).

^{13}C NMR (101 MHz, $CDCl_3$) δ = 155.9, 146.0, 141.2, 114.3, 112.3, 111.2, 79.0, 46.3, 25.9, 25.8, 18.3, 18.3, 16.1, -4.3, -4.5, -5.0.

$[\alpha]_D$ = -29.2 (c 2.6, CH_2Cl_2).

HRMS: (ESI+) calc. for $C_{29}H_{57}O_3Si_3$: 537.3612, found: 537.3616.

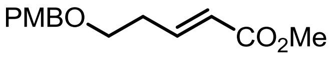

Trimethyl phosphonoacetate (5.46 g, 4.9 mL, 30 mmol) was added to a solution of K_2CO_3 (8.3 g, 60 mmol) in water (8.3 mL), cooled at 0 °C. The reaction mixture was stirred for 15 min, and a solution of the 3-(4-methoxybenzyloxy)propionaldehyde[1] (4.9 g, 25 mmol) in diethyl ether (10 mL) was then added. The heterogeneous mixture was stirred overnight at room temperature and then extracted with diethyl ether. The organic phase was dried with anhydrous Na_2SO_4 and concentrated to give (*E*)-methyl 5-(4-methoxybenzyloxy)pent-2-enoate (6.1 g, 98%) as a yellow oil, which was used in the next step without any further purification.

The analytical data are in accordance with those reported in the literature.[2]

[1] Herb, C.; Maier M. E. *J. Org. Chem.* **2003**, *68*, 8129-8135.

[2] Dias, L. C.; Sant'Ana, D. P.; Vieira, Y. W.; Gonçalves, C. C. S.; Lima D. J. P. *J. Braz. Chem. Soc.*, **2012**, *23*, 344-348.

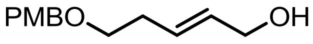

To a stirred solution of the above ester (6.1 g, 25 mmol) in CH_2Cl_2 (80 mL) at −78 °C was added dropwise DIBAL-H (1.2 M solution in toluene, 46 mL, 55 mmol). After 45 minutes the reaction mixture was quenched by pouring into a rapidly stirred mixture of 1 N HCl (400 mL) and CH_2Cl_2 (100 mL). The layers were separated and the aqueous layer was extracted with CH_2Cl_2 (50 mL). The combined organic layers were washed with 1 N HCl (100 mL), sat. aq. $NaHCO_3$ (50 mL), brine, dried over Na_2SO_4 and concentrated in vacuo. The crude product was purified by column chromatography on SiO_2 with petroleum ether/EtOAc 2:1 to give the allylic alcohol (5.3 g, 97%) as a colorless oil.

The analytical data are in accordance with those reported in the literature.[2]

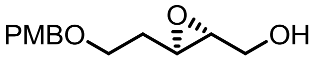

12

((2*R*,3*R*)-3-(2-(4-methoxybenzyloxy)ethyl)oxiran-2-yl)methanol (**12**)

To a suspension of activated 4 Å MS (5.1 g) in CH_2Cl_2 (120 mL) cooled at -20 °C was added *R*-(−)-diethyltartrate (0.6 mL, 3.4 mmol) followed by $Ti(OiPr)_4$ (1 mL, 3.4 mmol) under nitrogen atmosphere. The suspension was stirred for 20 min at −20 °C and then the above allylic alcohol (5.3 g, 24 mmol) in anhydrous dichloromethane (15 mL) was added dropwise and reaction mixture was stirred at same temperature for 30 min. TBHP (5.5 M in nonane, 8.7 mL, 48 mmol) was added and resulting mixture was stirred for 16 h at −20 °C. The reaction was quenched with water (15 mL) and stirred for 30 min at room temperature. 30% Aqueous NaOH solution saturated with NaCl (20 mL) was then added and the resulting mixture stirred vigorously for another 30 min. The reaction mixture was filtered through a short pad of Celite and washed with dichloromethane (2 × 30 mL). The layers were separated, the organic phase was dried over Na_2SO_4, the solvent was concentrated under reduce pressure and the residue thus obtained was purified by column chromatography over SiO_2 with petrol ether/EtOAc 2:1 to afford the epoxide **12** (4.6 g, 80%) as a viscous oil.

The analytical data match those reported for the enantiomer.[2]

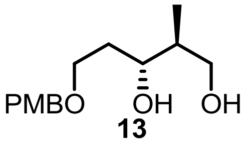

13

(2*S*,3*R*)-5-(4-methoxybenzyloxy)-2-methylpentane-1,3-diol (**13**)

To a suspension of purified[3] $CuBr{\bullet}Me_2S$ (150 mg, 0.71 mmol) in THF (25 mL) at −20 °C was added methylmagnesium bromide (3 M in Et_2O, 7 mL, 21 mmol) dropwise. After 30 min, the reaction mixture was cooled to −40 °C and a solution of epoxy alcohol **12** (1.7 g, 7 mmol) in

[3] Organic Syntheses, **2004**, Coll. Vol. 10, p.662; **2002**, Vol. 79, p.11.

Et_2O (10 mL) was added dropwise. The reaction mixture then allowed to warm to −20 °C during 1 h, stirred for further 6 h, slowly warmed to rt (over 3 h) and stirred at this temperature for 1 h. The reaction was then quenched with sat. aq. NH_4Cl (50 mL) at 0 °C and extracted with EtOAc (3 × 25 mL). The combined organic phases were evaporated under reduced pressure, the residue was dissolved in 1:1 acetone:water (20 mL), $NaIO_4$ (0.75 g, 3.5 mmol, 0.5 equiv) was added at 0 °C and the reaction was stirred at rt for 30 min. The reaction was quenched with sat. aq $NaHSO_3$ (20 mL), extracted with EtOAc (3 × 10 mL), washed with water and brine (20 mL), concentrated under reduced pressure and purified by column chromatography petroleum ether/EtOAc 3:2 to give diol **13** (1.4 g, 76%) as a colorless oil.

^{1}H NMR (300 MHz, $CDCl_3$) δ = 7.23 (d, *J*=8.7 Hz, 2H), 6.87 (d, *J*=8.7 Hz, 2H), 4.44 (s, 2H), 3.79 (s, 3H), 3.77 - 3.55 (m, 5H), 1.87 - 1.65 (m, 5H), 0.84 (d, *J*=7.0 Hz, 3H).

^{13}C NMR (75 MHz, $CDCl_3$) δ = 159.4, 129.7, 129.4, 114.0, 78.0, 73.2, 69.4, 67.8, 55.3, 40.2, 34.3, 13.9.

$[\alpha]_D$ = +78.6 (c 0.7, CH_2Cl_2)

HRMS (ESI^+) calc. for $C_{14}H_{22}O_4Na$: 277.1410, found 277.1408

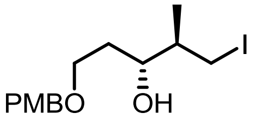

To a solution of diol **13** (1.3 g, 5.3 mmol, 1.0 equiv) in toluene (60 mL) at 0 °C was added triphenylphosphine (2.1 g, 8 mmol, 1.5 equiv) and pyridine (1.3 mL, 1.25 g, 16 mmol, 3.1 equiv). To this mixture was added a solution of I_2 (1.88 g, 7.42 mmol, 1.4 equiv) in toluene (30 mL) over 1.5 h, before warming to rt and stirring for 16 h. Cold hexane (100 mL) was added, and after 30 min, the reaction mixture was filtered through Celite and concentrated in vacuo. Flash chromatography (20% EtOAc/PE) over SiO_2 afforded the intermediate iodide (1.8 g, 100%) as a colourless oil.

^{1}H NMR (300 MHz, $CDCl_3$) δ = 7.23 (d, *J*=8.3 Hz, 2H), 6.87 (d, *J*=8.5 Hz, 2H), 4.50 - 4.38 (m, 2H), 3.78 (s, 3H), 3.75 - 3.52 (m, 3H), 3.42 - 3.32 (m, 2H), 1.86 - 1.65 (m, 2H), 1.51 - 1.37 (m, 1H), 0.95 (d, *J*=6.6 Hz, 3H)

^{13}C NMR (75 MHz, $CDCl_3$) δ = 159.4, 129.8, 129.4, 113.9, 74.8, 73.1, 69.2, 55.3, 40.3, 33.1, 17.4, 14.7.

HRMS (ESI^+) calc. for $C_{14}H_{21}IO_3Na$: 386.0349, found: 386.0351.

$[\alpha]_D$ = -20.2 (c 4.2, CH_2Cl_2)

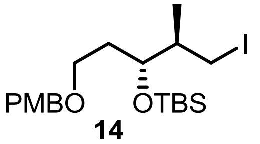

14

tert-Butyl((2*R*,3*R*)-1-iodo-5-(4-methoxybenzyloxy)-2-methylpentan-3-yloxy)dimethylsilane (**14**)

To a stirred solution of foregoing alcohol (1.8 g, 5.3 mmol) and 2,6-lutidine (1.2 mL, 10 mmol mmol) in CH_2Cl_2 (15 mL) at 0 °C was added dropwise *tert*-butyldimethylsilyl triflate (TBSOTf, 2.3 mL, 10 mmol) and the resulted mixture was stirred at 0 °C for 1 h. The reaction was quenched with water (25 mL), organic phase was separated, and the water phase extracted with CH_2Cl_2 (3 mL). Combined organic layers were washed with 1 N HCl (2 × 20 mL), sat. aq. $NaHCO_3$ (25 mL), brine (25 mL), and dried over anhydrous sodium sulfate. The solvent was removed in vacuo, and the crude product was purified by column chromatography over SiO_2 using petroleum ether/ethyl acetate 96:4 as an eluent to afford iodide **14** (2.3 g, 90%) as colorless oil.

^{1}H NMR (400 MHz, $CDCl_3$) δ = 7.25 (d, *J*=8.6 Hz, 2H), 6.87 (d, *J*=8.6 Hz, 2H), 4.45 - 4.38 (m, 2H), 3.80 (s, 3H), 3.78 - 3.74 (m, 1H), 3.51 (dt, *J*=2.8, 6.7 Hz, 2H), 3.22 (dd, *J*=5.1, 9.7 Hz, 1H), 3.10 (dd, *J*=7.1, 9.7 Hz, 1H), 1.80 - 1.67 (m, 3H), 0.99 (d, *J*=6.6 Hz, 3H), 0.86 (s, 9H), 0.07 (s, 3H), 0.04 (s, 3H)

^{13}C NMR (101 MHz, $CDCl_3$) δ = 159.2, 130.6, 129.3, 113.8, 72.7, 72.3, 66.5, 55.3, 40.9, 32.7, 25.9, 18.1, 16.4, 12.7, -4.4, -4.4.

HRMS (ESI$^+$) calcd for $C_{20}H_{35}ISiO_3Na$: 501.1299, found: 501.1298.

$[\alpha]_D$ = 2.0 (c 3.0, CH_2Cl_2)

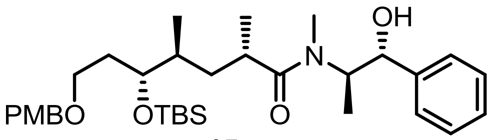

15

(2*S*,4*S*,5*R*)-5-(*tert*-butyldimethylsilyloxy)-N-((1*R*,2*R*)-1-hydroxy-1-phenylpropan-2-yl)-7-(4-methoxybenzyloxy)-*N*,2,4-trimethylheptanamide (**15**)

n-BuLi (2.5 M in hexanes, 6 mL, 14 mmol) was added to a suspension of LiCl (2 g, 50 mmol, dried for 18 h at 130 °C under high vacuum) and diisopropylamine (2 mL, 14 mmol) in THF (20 mL) at –78 °C. The resulting suspension was warmed to 0 °C for 10 min and cooled to −78 °C again. An ice-cooled solution of (*R*,*R*)-pseudoephedrine propionamide (1.55 g, 7 mmol) in THF (5 mL) was added at –78 °C and the mixture was stirred for one hour at –78 °C, 15 min at 0 °C, and 5 min at room temperature. To this solution iodide **13** (478 mg, 1 mmol) was added at 0 °C, and the reaction mixture was stirred at room temperature for 14 h. The yellow reaction mixture was treated with half-saturated aqueous NH_4Cl (50 mL) and extracted with EtOAc (2 × 50 mL). The combined organic layers were dried over Na_2SO_4 and

concentrated in vacuo. Flash chromatography over SiO_2 with Et_2O yielded amide **15** (565 mg, 95% yield) as a yellow oil.

^1H NMR (400 MHz, $CDCl_3$) δ = 7.36 - 7.20 (m, 9H), 4.46 - 4.33 (m, 3H), 3.82 - 3.77 (m, 4H), 3.53 - 3.42 (m, 4H), 2.79 (s, 3H), 1.64 - 1.56 (m, 4H), 1.07 (d, *J*=6.6 Hz, 4H), 1.02 (d, *J*=6.6 Hz, 3H), 0.85 (s, 9H), 0.82 (d, *J*=6.6 Hz, 3H), 0.00 (s, 3H), -0.01 (s, 3H)

^{13}C NMR (101 MHz, $CDCl_3$) δ = 179.3, 159.2, 142.5, 130.7, 129.3, 128.4, 127.7, 126.4, 113.8, 72.7, 72.6, 67.2, 55.3, 36.5, 36.0, 34.1, 32.0, 25.9, 18.1, 16.8, 14.5, 14.0, -4.3, -4.6.

HRMS: calc. for $C_{33}H_{53}NO_5SiNa$ 594.3580, found 594.3591.

$[\alpha]_D$ = -31.0 (c 1.9, CH_2Cl_2)

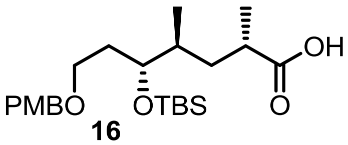

16

(2*S*,4*S*,5*R*)-5-(*tert*-butyldimethylsilyloxy)-7-(4-methoxybenzyloxy)-2,4-dimethylheptanoic acid (**16**)

A 10-mL round-bottomed flask was charged with amide **15** (547 mg, 0.95 mmol, 1 equiv), *tert*-butyl alcohol (10 mL), methanol (3 mL), and 4 N aqueous sodium hydroxide solution (3 mL, 12 mmol, 12 equiv). The mixture was stirred at 60 °C for 4 days and then cooled to rt. The mixture was poured into a stirred mixture of 1 M aqueous HCl (20 mL) and dichloromethane (20 mL). The aqueous layer was separated and extracted with dichloromethane (2 × 10 mL). The combined organic extracts were washed with brine, dried over sodium sulfate and concentrated in vacuo. Flash chromatography with petroleum ether/EtOAc 3:1 on silica afforded acid **16** (404 mg, 100%) as a colorless oil.

^1H NMR (300 MHz, $CDCl_3$) δ = 7.25 (d, *J*=8.5 Hz, 2H), 6.86 (d, *J*=8.7 Hz, 2H), 4.42 (d, *J*=11.5 Hz, 1H), 4.40 (d, *J*=11.5 Hz, 1H), 3.79 (s, 3H), 3.78 - 3.70 (m, 1H), 3.55 - 3.42 (m, 2H), 2.59 - 2.37 (m, 1H), 1.72 - 1.32 (m, 5H), 1.12 (d, *J*=6.8 Hz, 3H), 0.93 - 0.79 (m, 13H), 0.01 (s, 6H)

^{13}C NMR (75 MHz, $CDCl_3$) δ = 183.0, 159.2, 130.7, 129.3, 113.8, 72.7, 72.5, 67.3, 55.3, 37.2, 36.5, 36.3, 32.1, 26.0, 18.1, 16.8, 14.0, -4.4, -4.6.

HRMS: calc. for $C_{23}H_{40}O_5SiNa$ 447.2533, found 447.2543.

$[\alpha]_D$ = 8.5 (c 9.0, CH_2Cl_2)

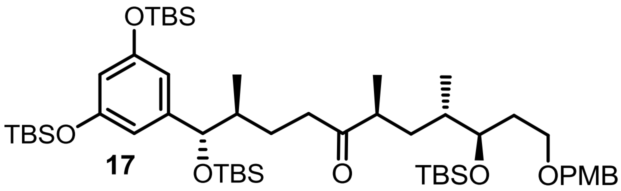

(5*R*,6*S*,8*S*,12*S*,13*S*)-13-(3,5-bis(*tert*-butyldimethylsilyloxy)phenyl)-5-(2-(4-methoxybenzyloxy)ethyl)-2,2,3,3,6,8,12,15,15,16,16-undecamethyl-4,14-dioxa-3,15-disilaheptadecan-9-one (**17**)

To a suspension of freshly prepared[4] Cp_2ZrHCl (0.6 mmol, 150 mg, 1.2 equiv) in THF (1.5 mL) was added a solution of alkene **10** (268 mg, 0.5 mmol) in THF (0.5 ml). The mixture was stirred at 40 °C for 45 min to assure complete consumption of Schwartz reagent. In parallel, a solution of carboxylic acid **16** (288 mg, 0.6 mmol) in CH_2Cl_2 (1 mL) was treated at 0 °C with freshly distilled or purchased 1-chloro-*N,N*,2-trimethyl-1-propenylamine (Ghosez reagent, 135 μL, 1 mmol). The solution was stirred for 30 min at 0 °C and 2 h at rt at which point all volatiles were removed in vacuo. The residue was dissolved in THF (1 mL) and the solvent was evaporated again. The acid chloride was transferred in THF (1 mL) to the solution of zirconium reagent via double tipped cannula following 0.3 ml rinse. Finally, freshly purified[3] $CuBr•Me_2S$ (20 mg, 0.1 mmol, 0.2 equiv) was added in one portion and the reaction mixture was stirred for 2 h at 40 °C. Upon completion reaction was quenched with aq. $NaHCO_3$ (5 mL) and filtered through a pad of Celite. The organic phase was separated, the aqueous layer extracted with EtOAc (5 mL), combined organic phases were washed with brine, dried over sodium sulfate and concentrated in vacuo. Flash chromatography with petroleum ether/EtOAc 12:1 on silica afforded ketone **16** (325 mg, 65%) as a colorless oil.

^{1}H NMR (400 MHz, $CDCl_3$) δ = 7.27 - 7.21 (m, 2H), 6.86 (d, *J*=8.6 Hz, 2H), 6.36 (d, *J*=2.0 Hz, 2H), 6.21 (t, *J*=2.0 Hz, 1H), 4.42 (d, *J*=11.7 Hz, 1H), 4.37 (d, *J*=11.7 Hz, 1H), 4.19 (d, *J*=6.6 Hz, 1H), 3.79 (s, 3H), 3.69 - 3.64 (m, 1H), 3.51 - 3.42 (m, 2H), 2.54 - 2.41 (m, 2H), 2.37 - 2.27 (m, 1H), 1.94 - 1.84 (m, 1H), 1.67 - 1.55 (m, 5H), 1.43 - 1.19 (m, 4H), 0.99 - 0.96 (m, 4H), 0.96 - 0.95 (m, 16H), 0.87 - 0.84 (m, 17H), 0.83 (d, *J*=6.6 Hz, 3H), 0.69 (d, *J*=7.1 Hz, 3H), 0.15 (s, 12H), 0.00 (s, 9H), -0.21 (s, 3H)

^{13}C NMR (75 MHz, $CDCl_3$) δ = 214.7, 159.2, 156.1, 146.3, 130.8, 129.3, 113.8, 112.4, 111.2, 79.4, 73.0, 72.7, 67.2, 55.3, 43.8, 41.2, 38.9, 36.3, 35.5, 32.3, 26.3, 26.0, 25.8, 18.3, 18.2, 18.2, 16.0, 15.8, 14.1, -4.3, -4.4, -4.5, -5.0.

HRMS: calc. for $C_{52}H_{96}O_7Si_4Na$ 967.6125, found 967.6131.

$[\alpha]_D$ = 10.6 (c 1.75, CH_2Cl_2)

[4] Huang, Z.; Negishi, E. *Org. Lett.*, **2006**, 8, 3675-3678.

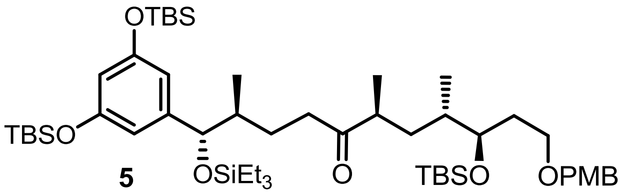

5

(5*R*,6*S*,8*S*,12*S*,13*S*)-13-(3,5-bis(*tert*-butyldimethylsilyloxy)phenyl)-15,15-diethyl-5-(2-(4-methoxybenzyloxy)ethyl)-2,2,3,3,6,8,12-heptamethyl-4,14-dioxa-3,15-disilaheptadecan-9-one (**5**)

Compound **5** was obtained with 50% yield from alkene **6** according to the previous procedure.

^{1}H NMR (500 MHz, $CDCl_3$) δ = 7.24 (d, *J*=8.9 Hz, 2H), 6.86 (d, *J*=8.7 Hz, 2H), 6.37 (d, *J*=2.1 Hz, 2H), 6.21 (t, *J*=2.2 Hz, 1H), 4.42 (d, *J*=11.4 Hz, 1H), 4.37 (d, *J*=11.4 Hz, 1H), 4.19 (d, *J*=6.9 Hz, 1H), 3.79 (s, 3H), 3.69 - 3.65 (m, 1H), 3.51 - 3.44 (m, 2H), 2.54 - 2.31 (m, 3H), 1.97 - 1.88 (m, 1H), 1.67 - 1.56 (m, 6H), 1.42 - 1.21 (m, 6H), 0.96 (s, 24H), 0.87 - 0.82 (m, 24H), 0.67 (d, *J*=6.7 Hz, 3H), 0.51 - 0.45 (m, 6H), 0.16 (s, 12H), 0.00 (s, 5H)

^{13}C NMR (126 MHz, $CDCl_3$) δ = 214.8, 159.1, 156.0, 146.4, 130.7, 129.3, 113.8, 112.4, 112.3, 111.2, 79.5, 73.0, 72.7, 67.2, 55.3, 43.8, 41.2, 39.0, 36.2, 35.5, 32.2, 26.6, 25.9, 25.8, 18.3, 18.1, 16.0, 15.8, 14.0, 6.9, 4.9, -4.3, -4.3, -4.6.

HRMS: calc. for $C_{52}H_{96}O_7Si_4Na$ 967.6125, found 967.6123.

$[\alpha]_D$ = 7.14 (c 4.3, CH_2Cl_2)

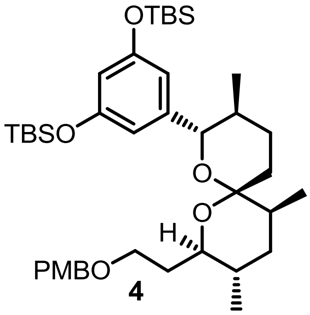

4

(5-((2*S*,3*S*,6*R*,8*R*,9*S*,11*S*)-8-(2-(4-methoxybenzyloxy)ethyl)-3,9,11-trimethyl-1,7-dioxaspiro[5.5]undecan-2-yl)-1,3-phenylene)bis(oxy)bis(tert-butyldimethylsilane) (**4**)

To a stirred solution of TBAF (80 mg) in THF (1 mL) was added acetic acid (120 μL, 2 mmol) followed by solution of ketone **17** (60 mg, 0.065 mmol) in THF (200 μL) and the mixture was stirred for 16 h. The reaction was quenched with sat. aq. $NaHCO_3$ (2 mL) and extracted with EtOAc (2 × 1 mL). The combined organic phases were washed with brine, dried over sodium sulfate and concentrated in vacuo. The residue was dissolved in in CH_2Cl_2 (1 mL) at 0 °C followed by addition of 2,6-lutidine (20 μL, 0.2 mmol) and *tert*-butyldimethylsilyl triflate (TBSOTf, 30 μL, 0.13 mmol) the resulted mixture was stirred at 0 °C for 1 h. The reaction

was quenched with water (1 mL), organic phase was separated, and the water phase extracted with CH_2Cl_2 (1 mL). Combined organic layers were washed with 1 N HCl (2 × 1 mL), sat. aq. $NaHCO_3$ (2 mL), brine (2 mL), and dried over anhydrous sodium sulfate. The solvent was removed in vacuo, and the crude product was purified by column chromatography over SiO_2 using petroleum ether/ethyl acetate 10:1 as an eluent to afford spiroketal **4** (16 mg, 35%) as colorless oil.

^{1}H NMR (300 MHz, $CDCl_3$) δ = 7.28 (d, *J*=8.7 Hz, 2H), 6.87 (d, *J*=8.7 Hz, 2H), 6.43 (d, *J*=2.3 Hz, 2H), 6.25 (t, *J*=2.3 Hz, 1H), 4.59 (d, *J*=11.7 Hz, 1H), 4.43 (d, *J*=11.7 Hz, 1H), 3.94 (d, *J*=9.8 Hz, 1H), 3.79 (s, 3H), 3.72 - 3.59 (m, 2H), 3.35 - 3.23 (m, 1H), 2.05 - 1.84 (m, 2H), 1.69 - 1.54 (m, 7H), 0.97 - 0.92 (m, 23H), 0.74 (d, *J*=6.0 Hz, 3H), 0.65 (d, *J*=6.6 Hz, 3H), 0.17 (s, 12H)

^{13}C NMR (75 MHz, $CDCl_3$) δ = 159.2, 156.0, 144.2, 130.7, 129.4, 113.9, 113.0, 111.3, 97.8, 78.1, 77.3, 72.8, 71.4, 67.3, 55.3, 38.9, 36.9, 36.6, 36.1, 33.4, 32.2, 28.3, 25.8, 18.3, 18.1, 17.5, 16.4, -4.3, -4.3.

HRMS: calc. for $C_{40}H_{66}O_6Si_2Na$ 721.4290, found 721.4288.

$[\alpha]_D$ =- 17.3 (c 1.6, CH_2Cl_2).
