# Supplementary material for: Synthesis of the spiroketal core of integramycin
Source: Beilstein J Org Chem. 2013 Nov 12;9:2446–50. doi: 10.3762/bjoc.9.282 (PMC3869345; doi:10.3762/bjoc.9.282)
Supplement: File 2 — Copies of NMR spectra. [file Beilstein_J_Org_Chem-09-2446-s002.pdf]

Supporting Information

for

Synthesis of the spiroketal core of integramycin

Evgeny. V. Prusov*

Address: Department of Medicinal Chemistry, Helmholtz Centre for Infection Research, Inhoffenstr. 7, 38124 Braunschweig, Germany

Email: Evgeny. V. Prusov - evgeny.prusov@helmholtz-hzi.de

*Corresponding author

Copies of NMR-spectra

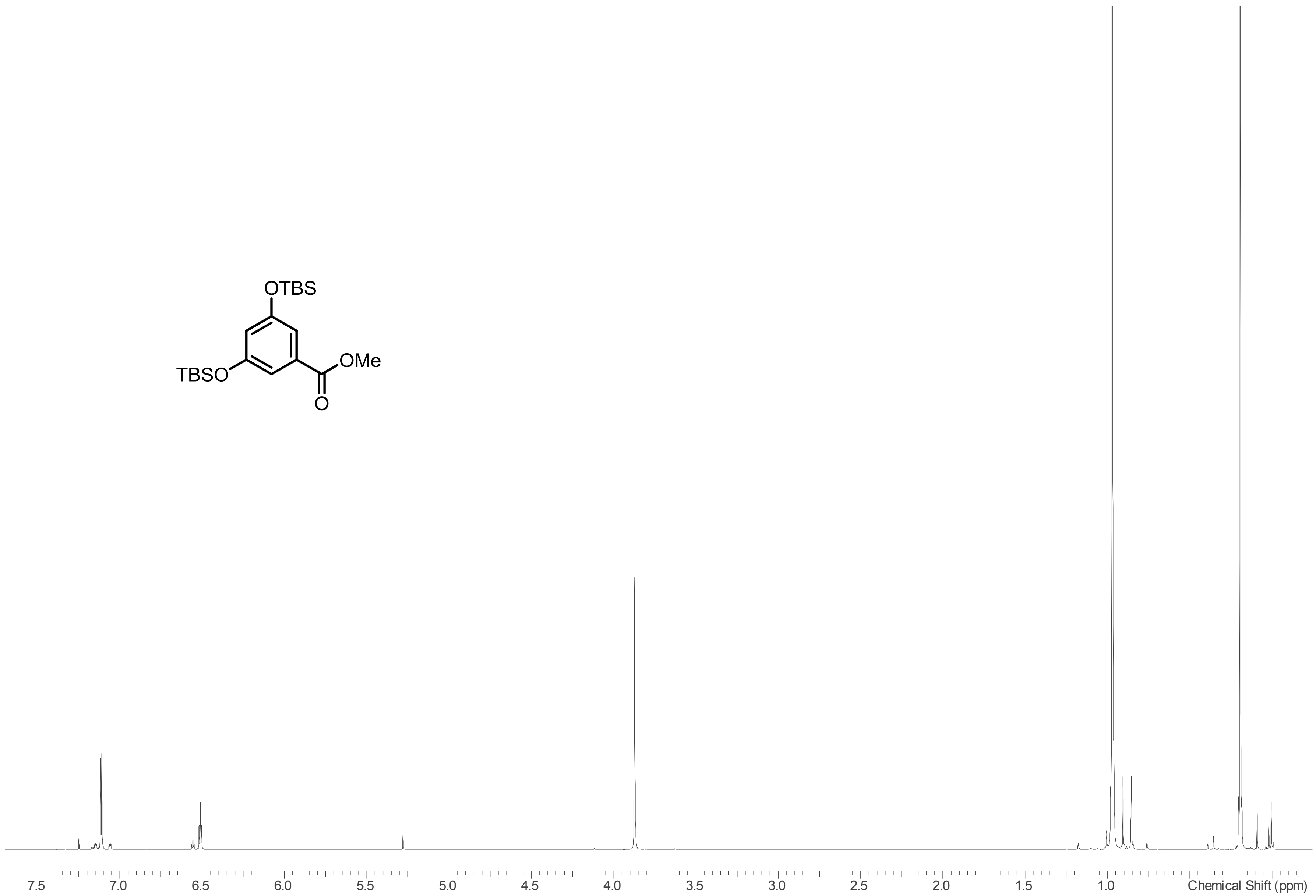
OTBS
TBSO
OMe
O
7.5
7.0
6.5
6.0
5.5
5.0
4.5
4.0
3.5
3.0
2.5
2.0
1.5
1.0
Chemical Shift (ppm)

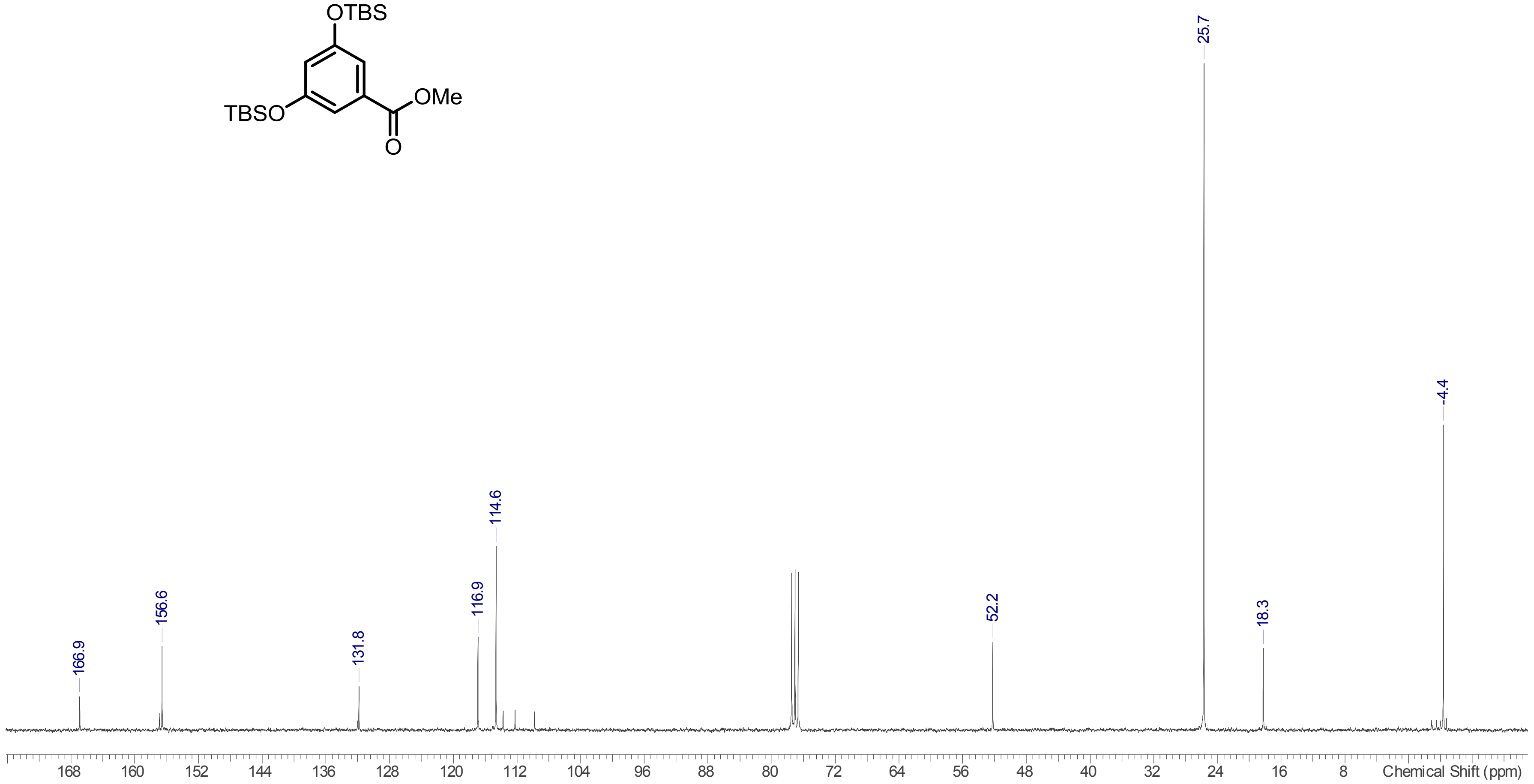
OTBS
TBSO
OMe
O
166.9
156.6
131.8
116.9
114.6
52.2
25.7
18.3
-4.4
168
160
152
144
136
128
120
112
104
96
88
80
72
64
56
48
40
32
24
16
8
Chemical Shift (ppm)

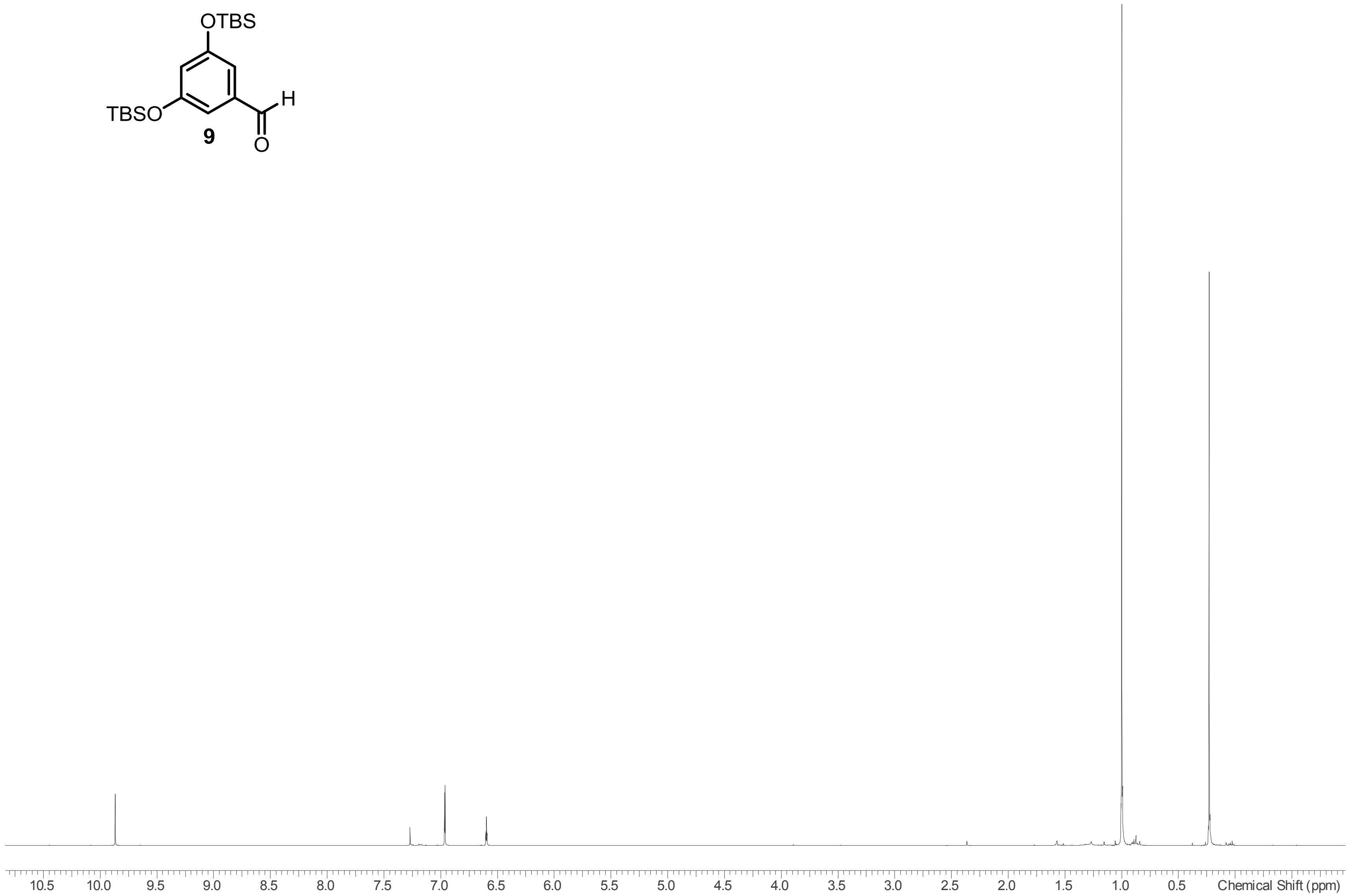
OTBS
TBSO
H
O
9
10.5
10.0
9.5
9.0
8.5
8.0
7.5
7.0
6.5
6.0
5.5
5.0
4.5
4.0
3.5
3.0
2.5
2.0
1.5
1.0
0.5
Chemical Shift (ppm)

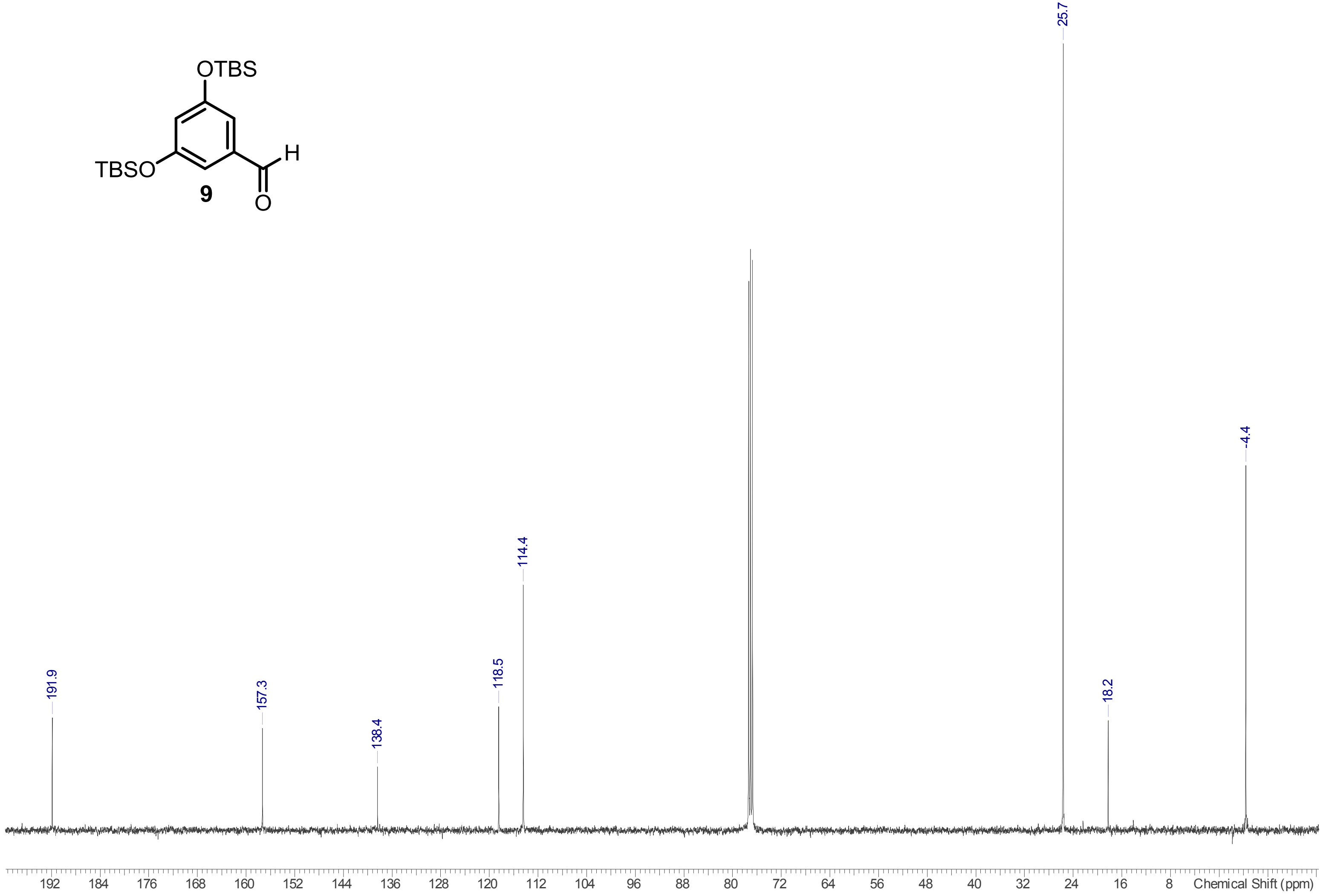
OTBS
TBSO
H
O
9
191.9
157.3
138.4
118.5
114.4
25.7
18.2
-4.4
192 184 176 168 160 152 144 136 128 120 112 104 96 88 80 72 64 56 48 40 32 24 16 8
Chemical Shift (ppm)

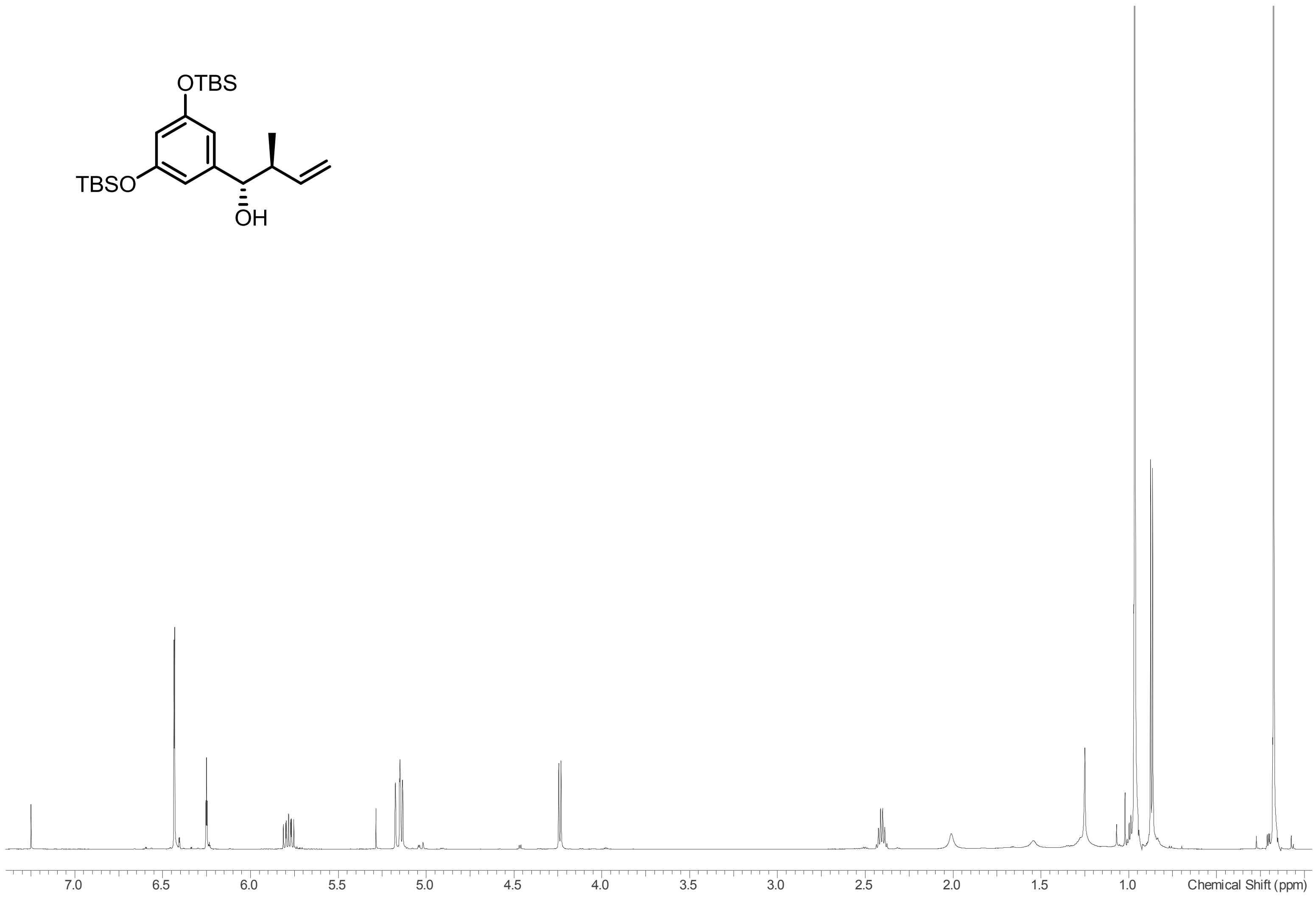
OTBS
TBSO
OH
7.0
6.5
6.0
5.5
5.0
4.5
4.0
3.5
3.0
2.5
2.0
1.5
1.0
Chemical Shift (ppm)

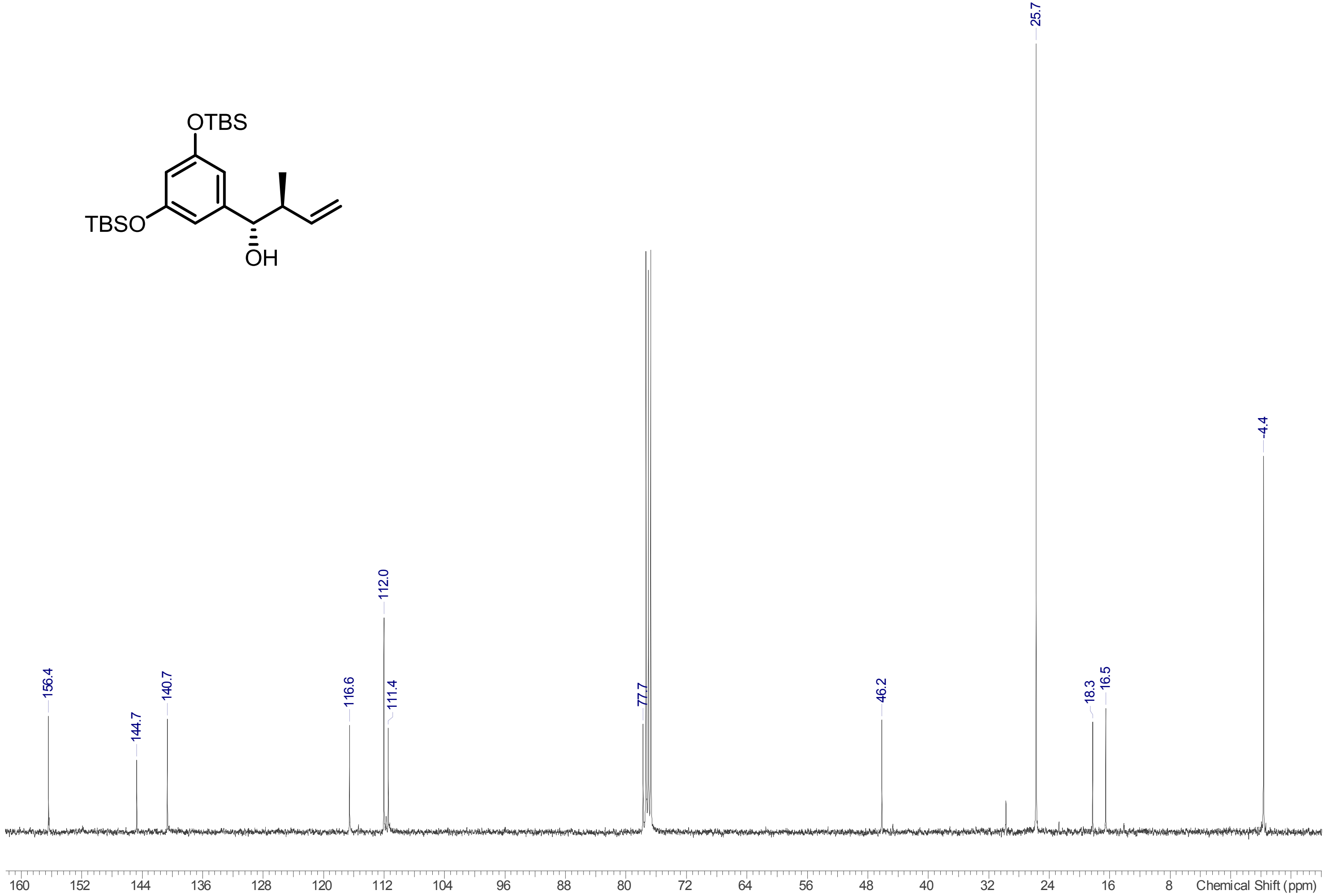
OTBS
TBSO
OH
25.7
-4.4
112.0
156.4
140.7
116.6
111.4
77.7
46.2
18.3
16.5
144.7
160
152
144
136
128
120
112
104
96
88
80
72
64
56
48
40
32
24
16
8
Chemical Shift (ppm)

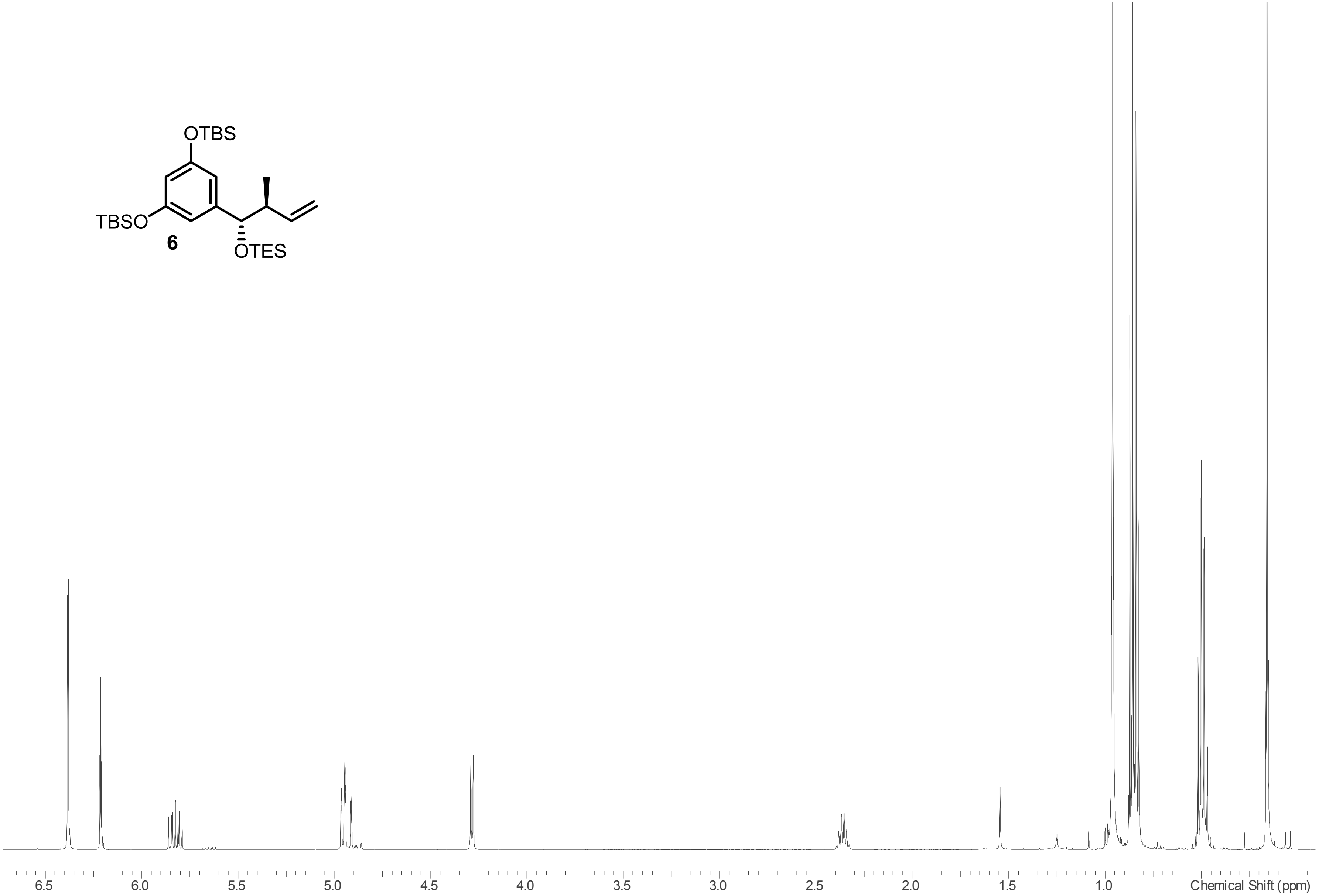
OTBS
TBSO
OTES
6
6.5
6.0
5.5
5.0
4.5
4.0
3.5
3.0
2.5
2.0
1.5
1.0
Chemical Shift (ppm)

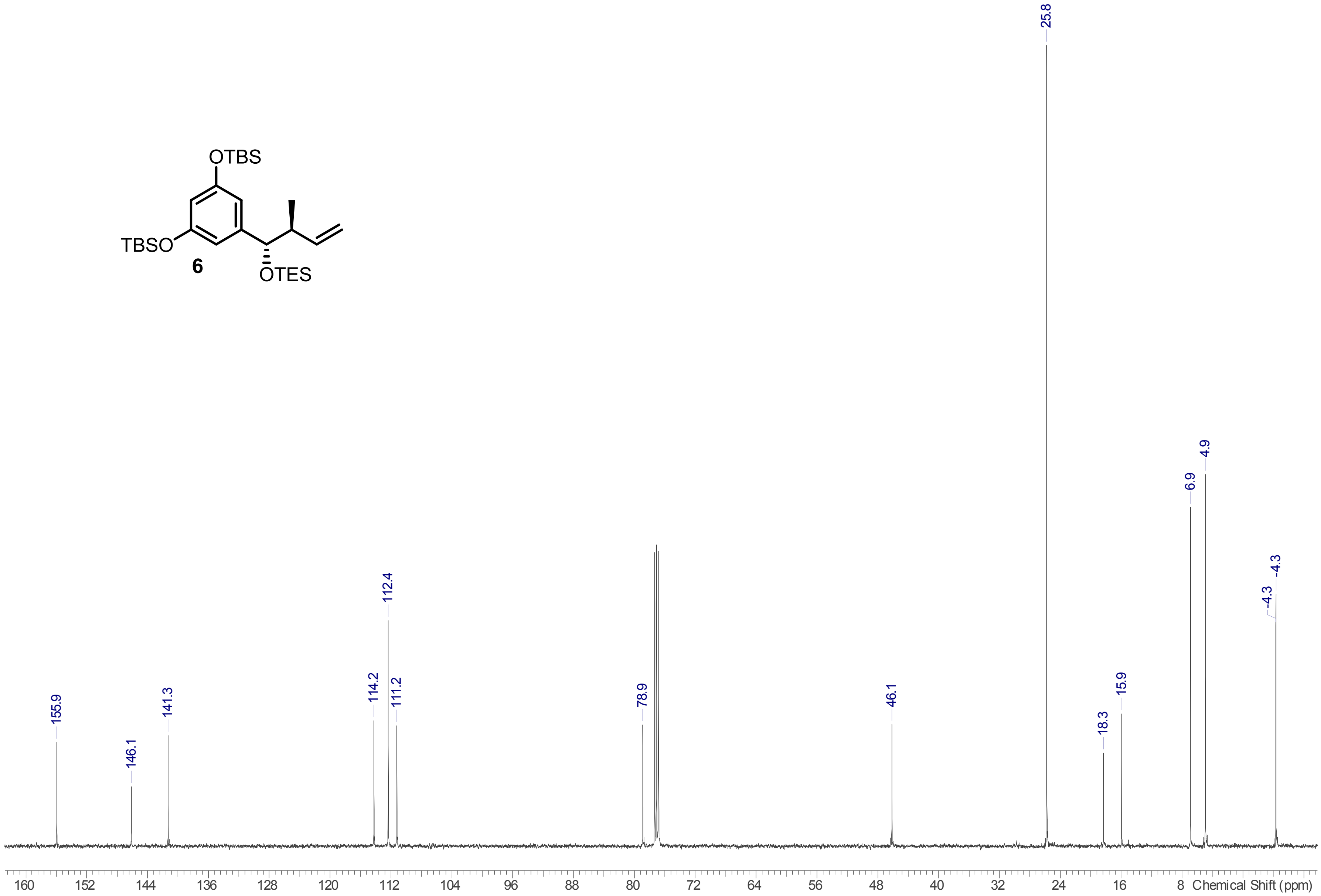

OTBS
TBSO
6
OTES
155.9
146.1
141.3
114.2
112.4
111.2
78.9
46.1
25.8
18.3
15.9
6.9
4.9
-4.3
-4.3
160
152
144
136
128
120
112
104
96
88
80
72
64
56
48
40
32
24
16
8
Chemical Shift (ppm)

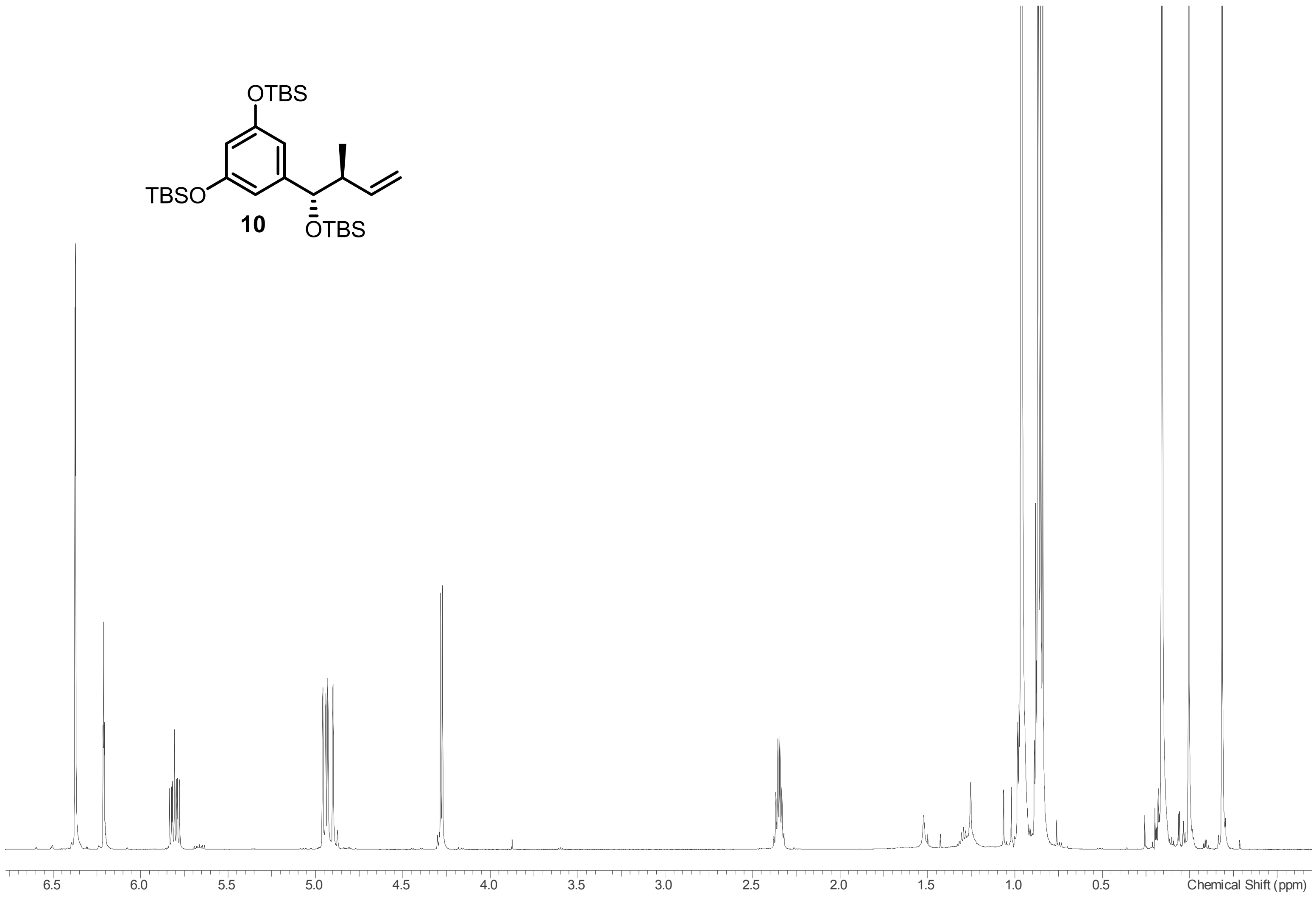
OTBS
TBSO
OTBS
10
6.5
6.0
5.5
5.0
4.5
4.0
3.5
3.0
2.5
2.0
1.5
1.0
0.5
Chemical Shift (ppm)

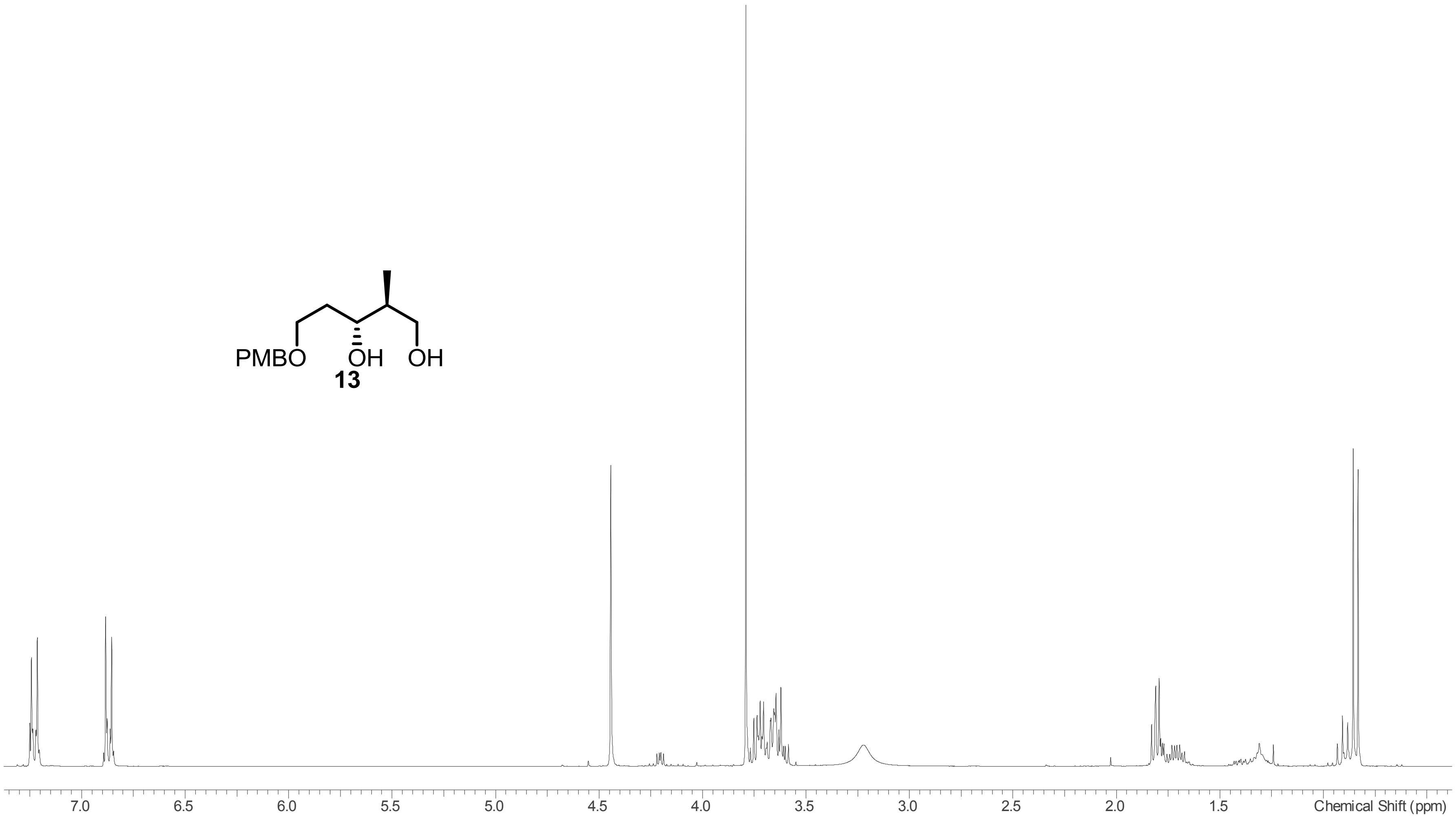
PMBO
OH
OH
13
7.0
6.5
6.0
5.5
5.0
4.5
4.0
3.5
3.0
2.5
2.0
1.5
Chemical Shift (ppm)

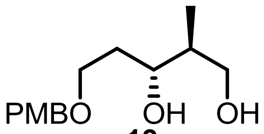
PMBO
OH
OH

13

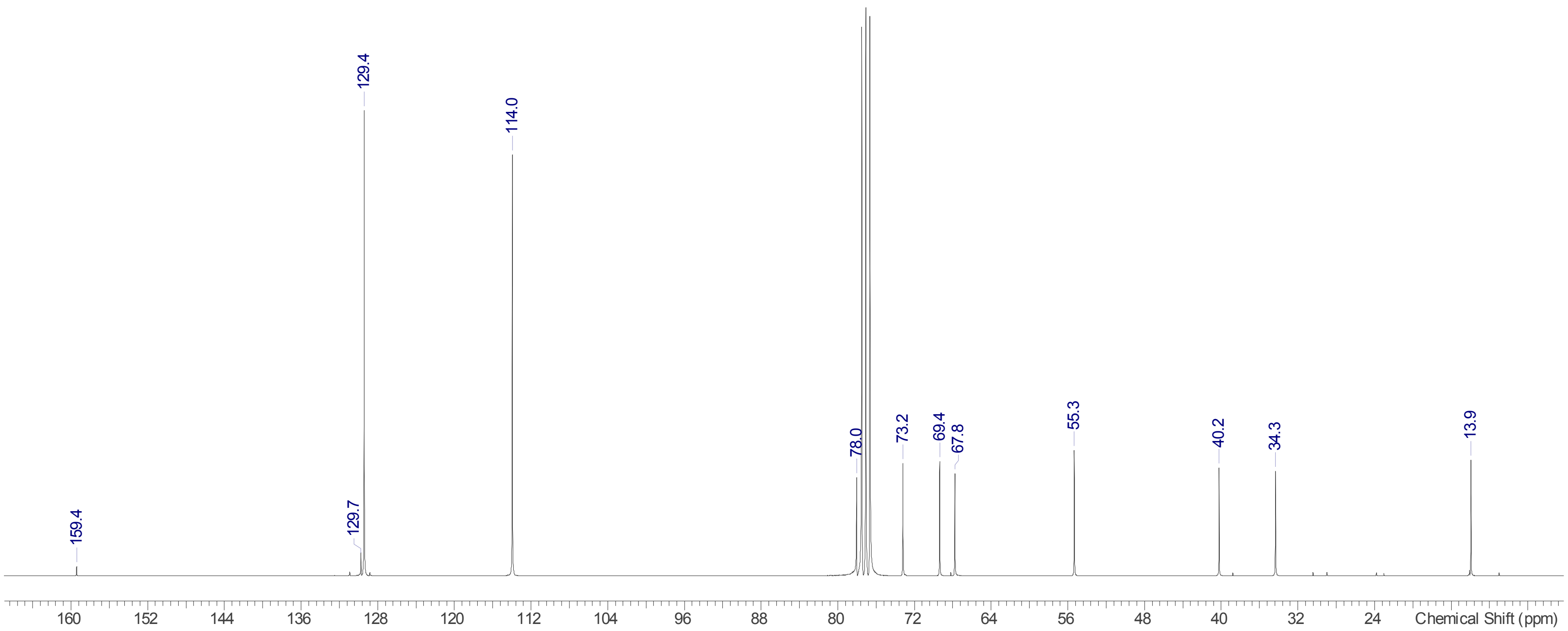
159.4
129.7
129.4
114.0
78.0
73.2
69.4
67.8
55.3
40.2
34.3
13.9
160
152
144
136
128
120
112
104
96
88
80
72
64
56
48
40
32
24
Chemical Shift (ppm)

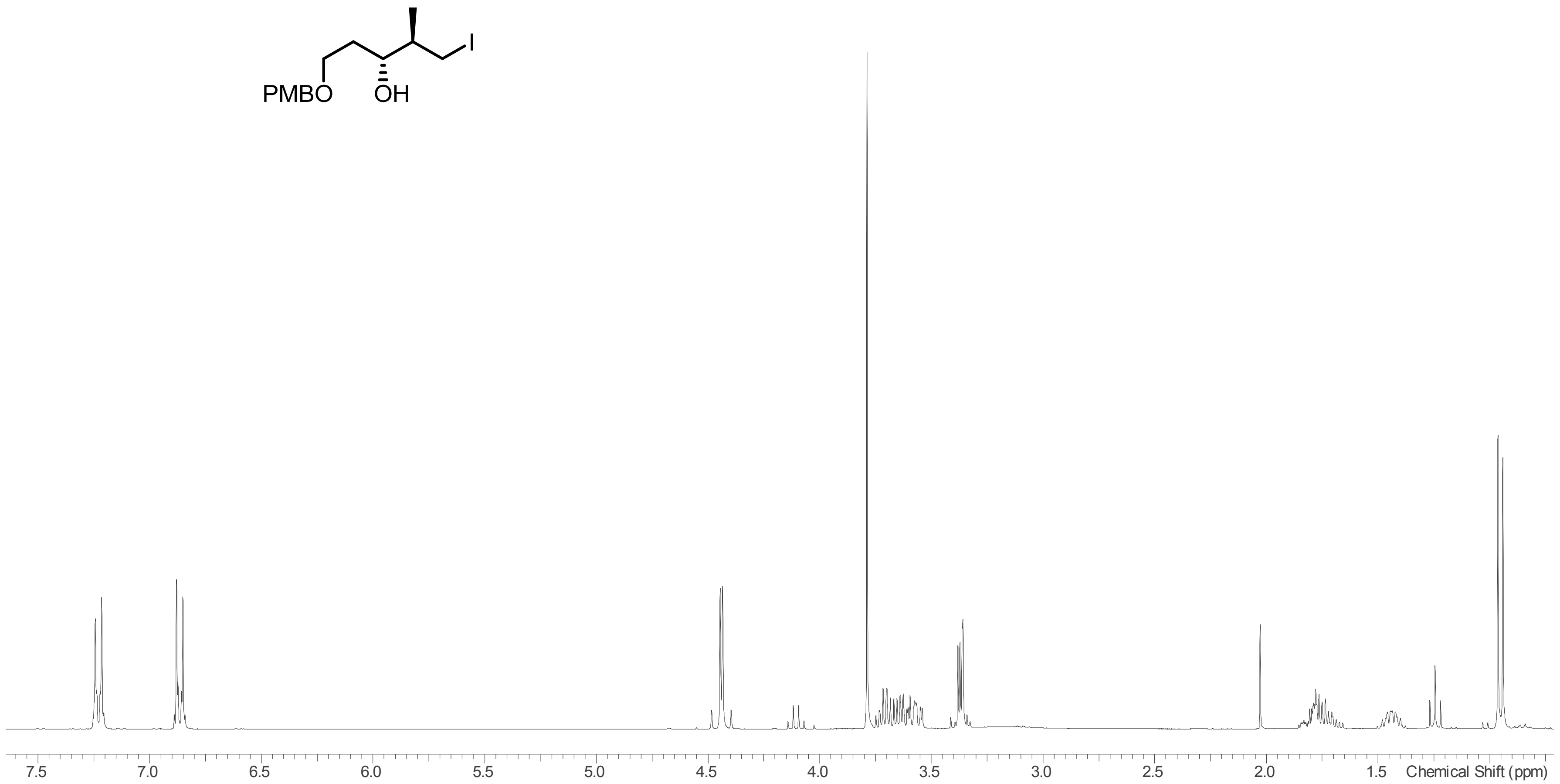
PMBO
OH
I
7.5
7.0
6.5
6.0
5.5
5.0
4.5
4.0
3.5
3.0
2.5
2.0
1.5
Chemical Shift (ppm)

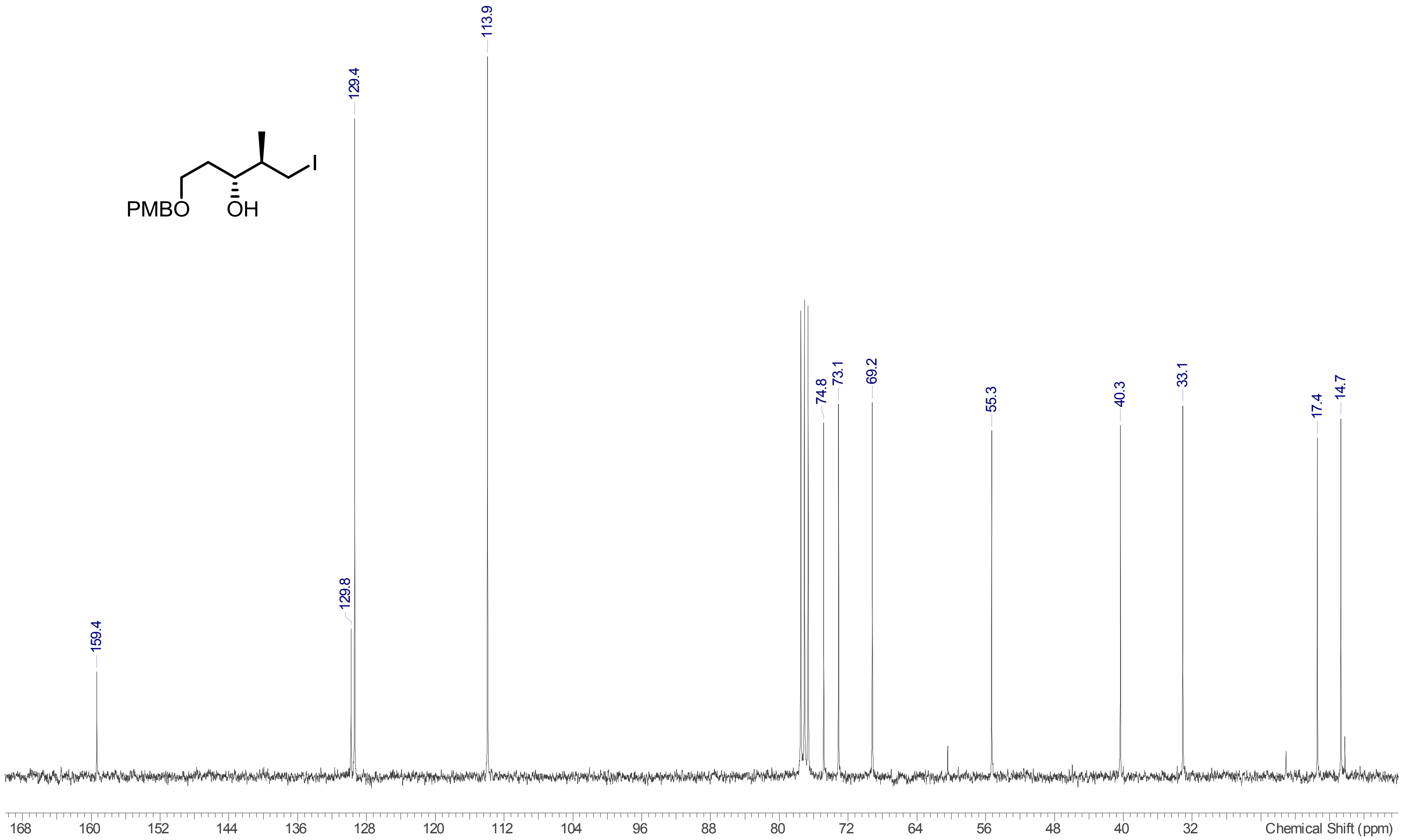

PMBO
OH
I
113.9
129.4
129.8
159.4
74.8
73.1
69.2
55.3
40.3
33.1
17.4
14.7
168
160
152
144
136
128
120
112
104
96
88
80
72
64
56
48
40
32
Chemical Shift (ppm)

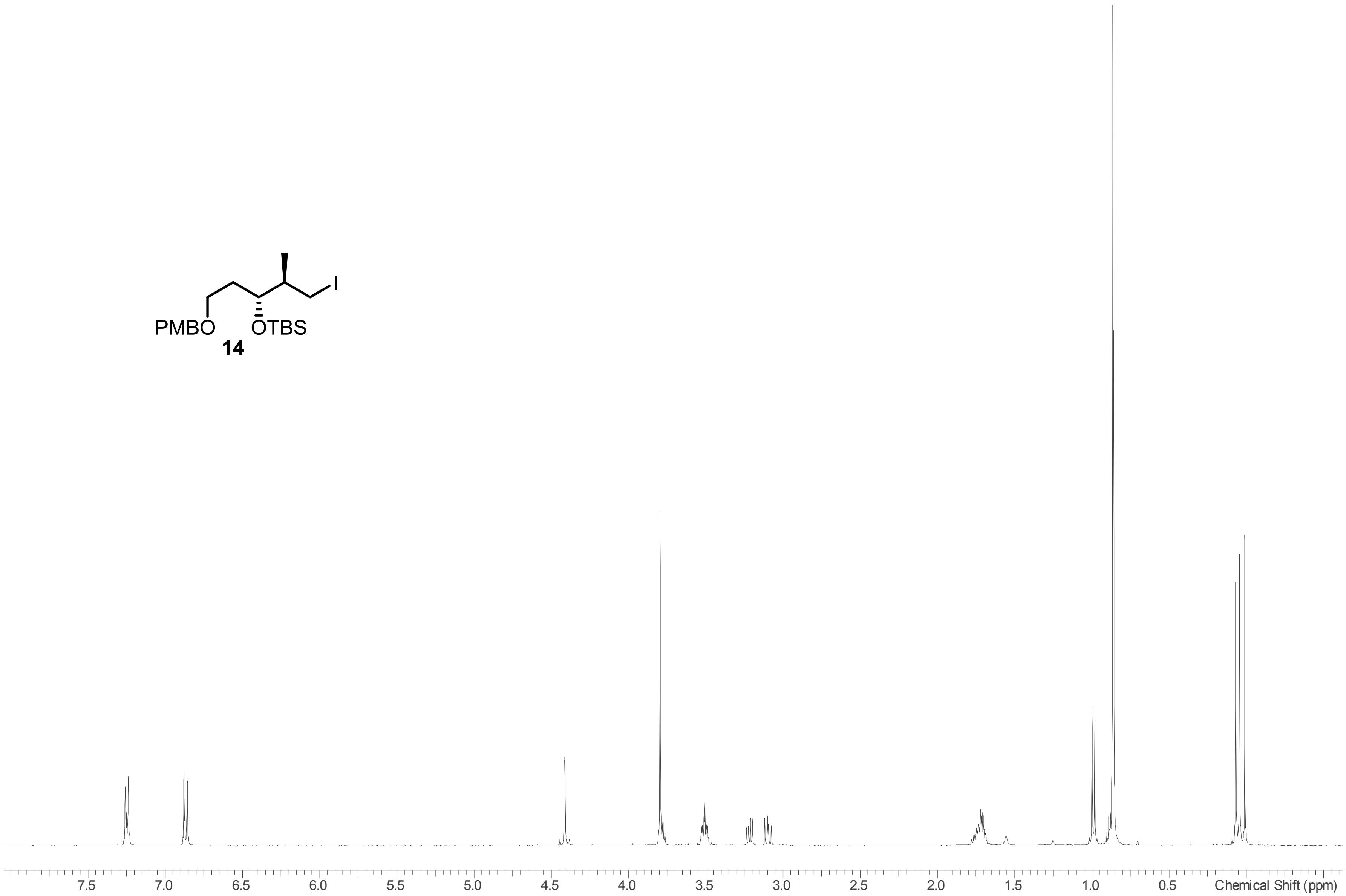
PMBO
OTBS
I
14
7.5
7.0
6.5
6.0
5.5
5.0
4.5
4.0
3.5
3.0
2.5
2.0
1.5
1.0
0.5
Chemical Shift (ppm)

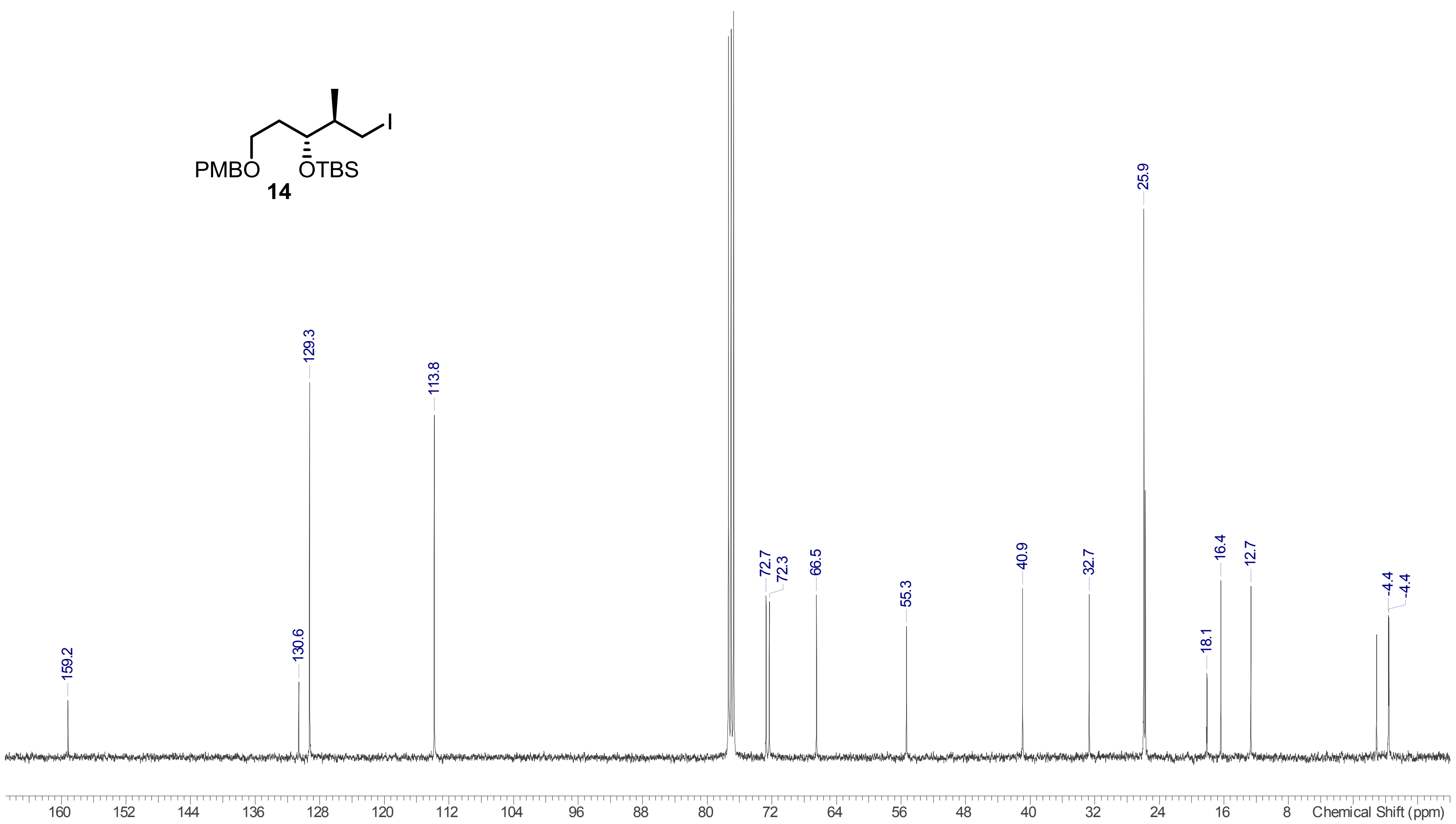
PMBO
OTBS
I
14
159.2
130.6
129.3
113.8
72.7
72.3
66.5
55.3
40.9
32.7
25.9
18.1
16.4
12.7
-4.4
-4.4
160
152
144
136
128
120
112
104
96
88
80
72
64
56
48
40
32
24
16
8
Chemical Shift (ppm)

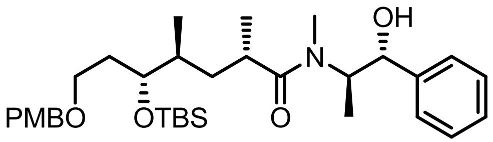
OH
N
PMBO
OTBS
O

15

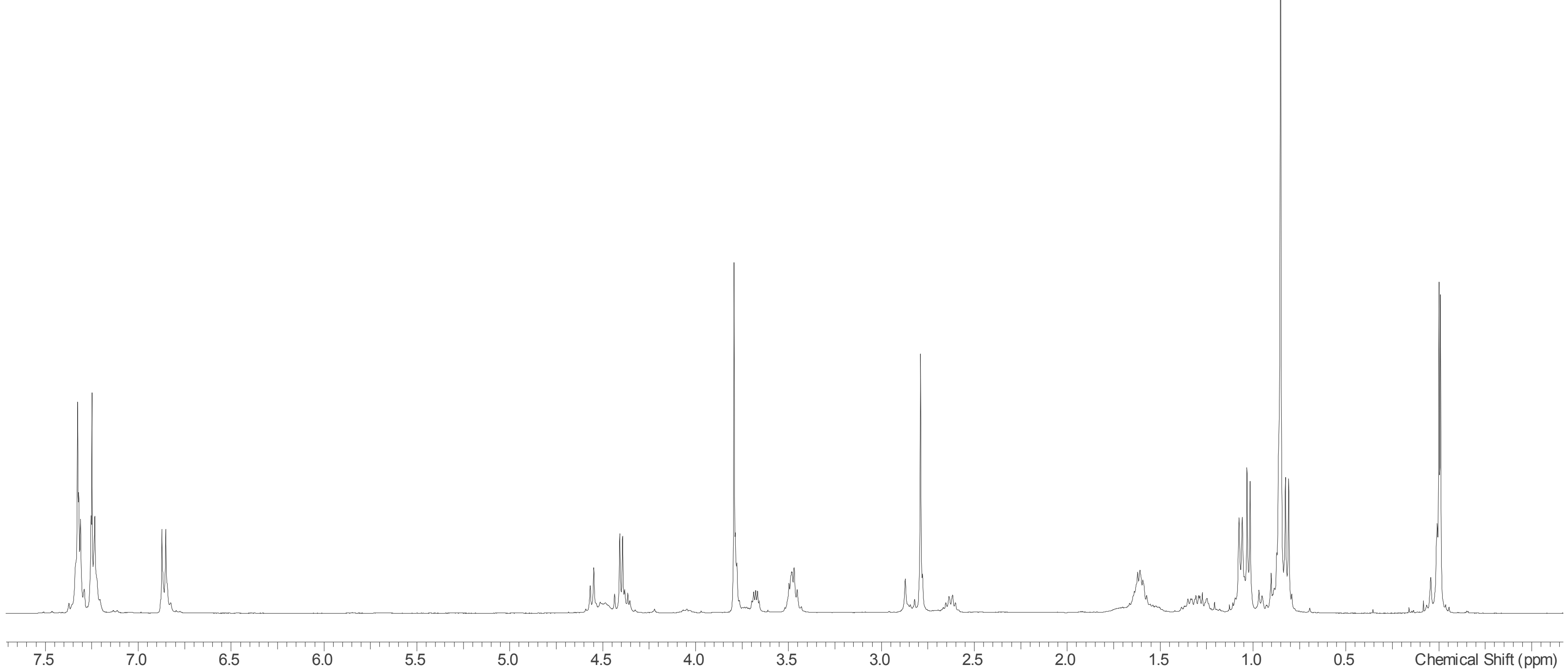
7.5
7.0
6.5
6.0
5.5
5.0
4.5
4.0
3.5
3.0
2.5
2.0
1.5
1.0
0.5
Chemical Shift (ppm)

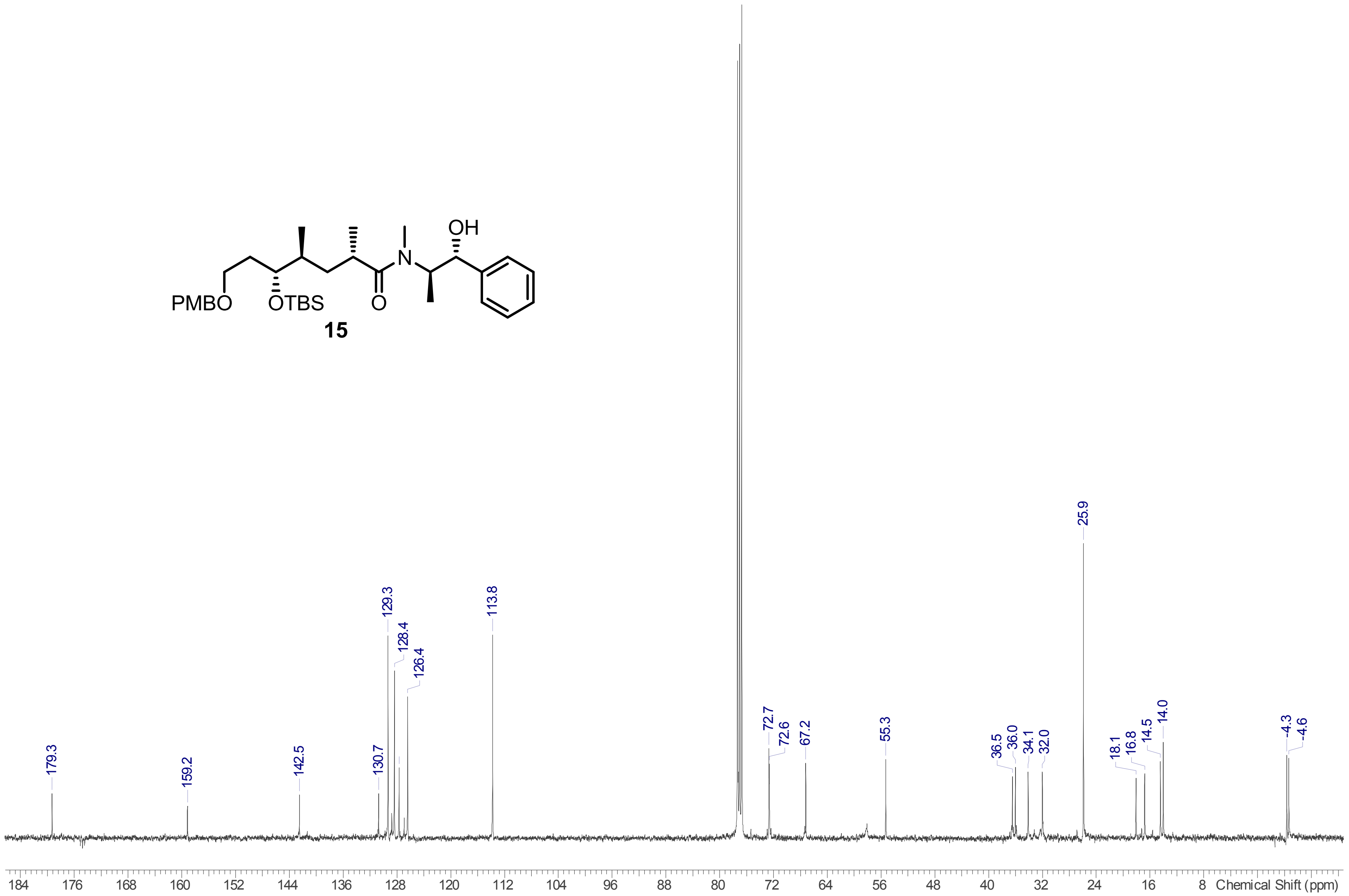
PMBO
OTBS
O
N
OH
15
179.3
159.2
142.5
130.7
129.3
128.4
126.4
113.8
72.7
72.6
67.2
55.3
36.5
36.0
34.1
32.0
25.9
18.1
16.8
14.5
14.0
-4.3
-4.6
184
176
168
160
152
144
136
128
120
112
104
96
88
80
72
64
56
48
40
32
24
16
8
Chemical Shift (ppm)

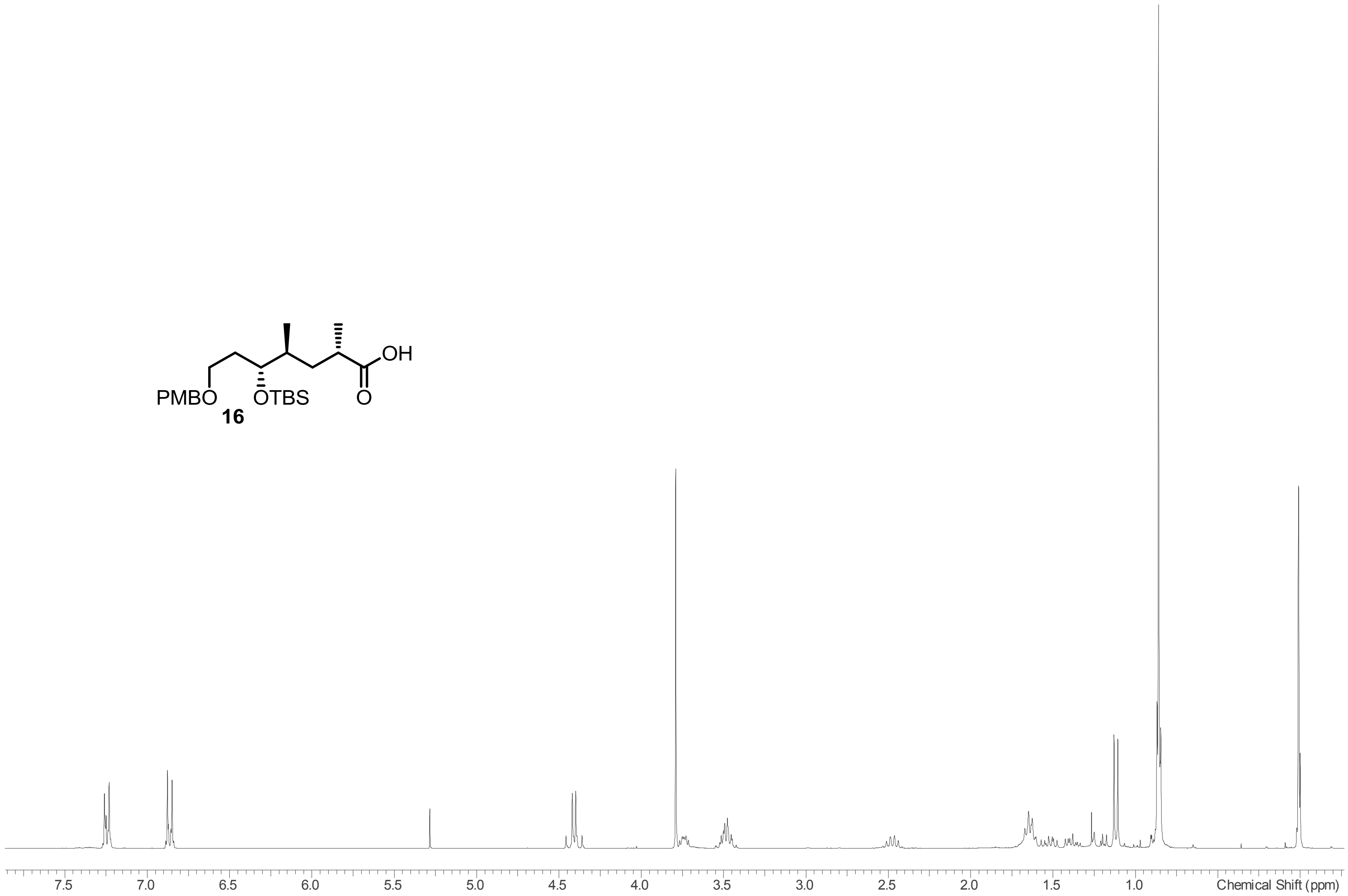

PMBO
OTBS
OH
O
16
7.5
7.0
6.5
6.0
5.5
5.0
4.5
4.0
3.5
3.0
2.5
2.0
1.5
1.0
Chemical Shift (ppm)

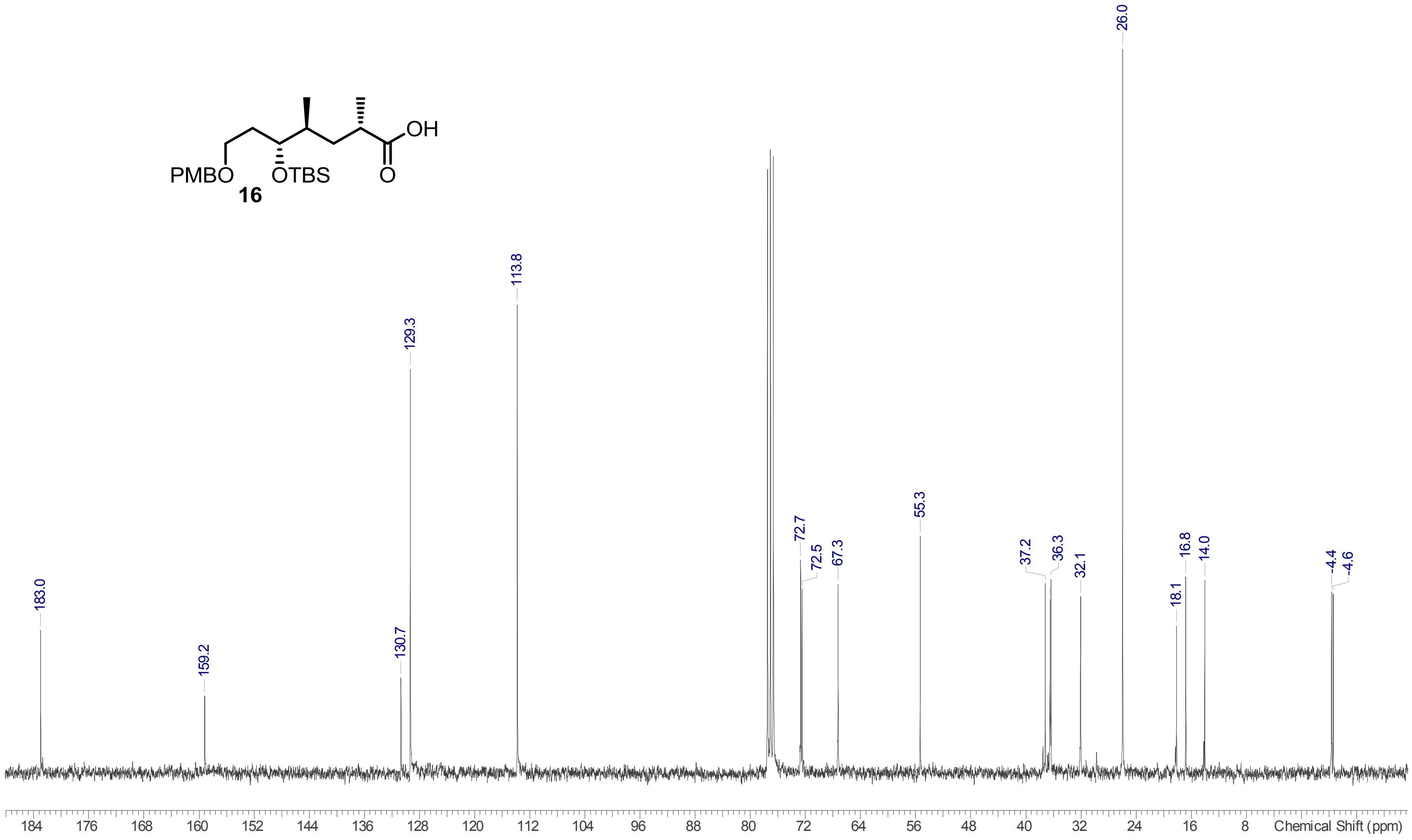
PMBO
OTBS
OH
O
16
183.0
159.2
130.7
129.3
113.8
72.7
72.5
67.3
55.3
37.2
36.3
32.1
26.0
18.1
16.8
14.0
-4.4
-4.6
184 176 168 160 152 144 136 128 120 112 104 96 88 80 72 64 56 48 40 32 24 16 8
Chemical Shift (ppm)

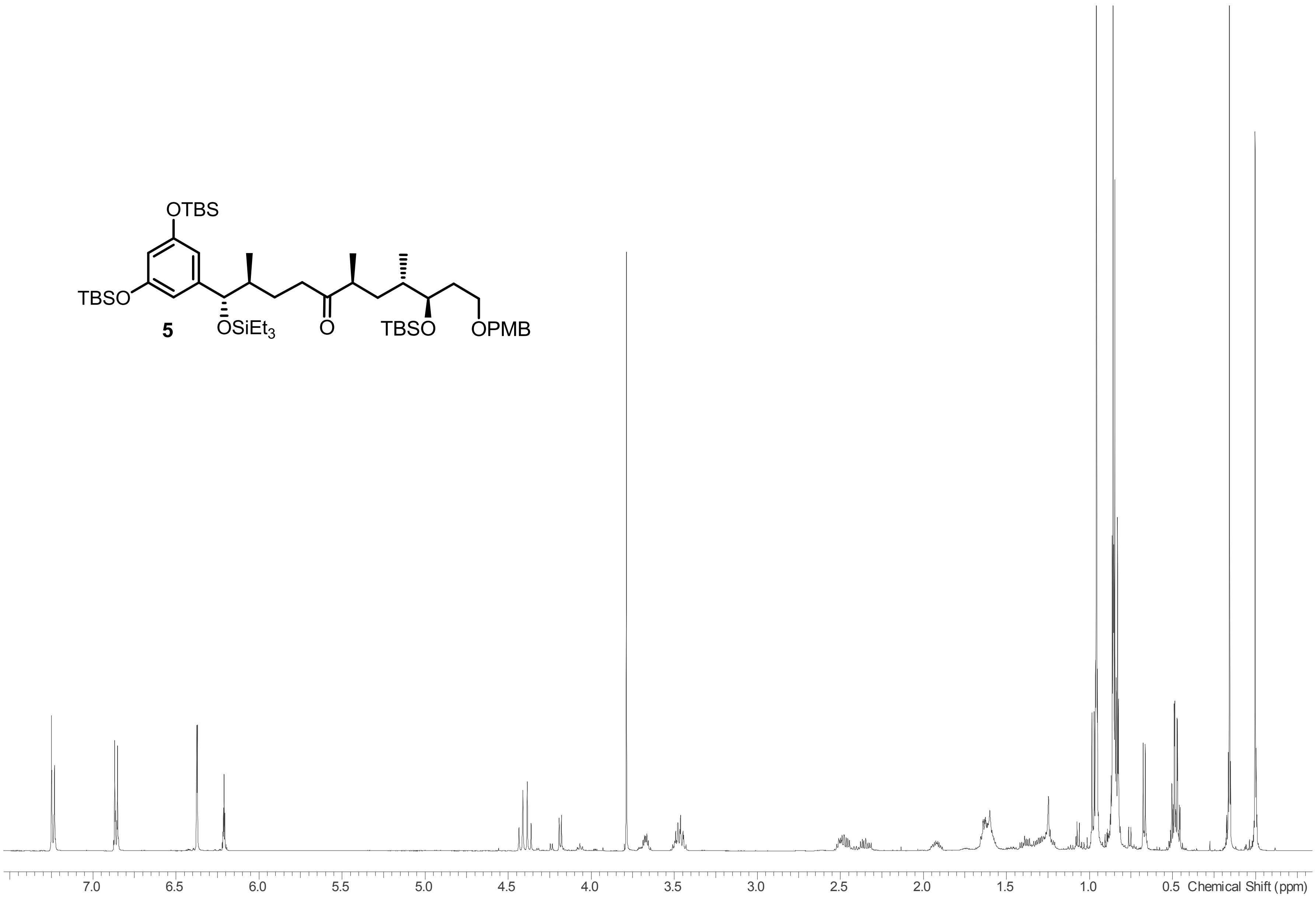
OTBS
TBSO
OSiEt3
O
TBSO
OPMB
5
7.0 6.5 6.0 5.5 5.0 4.5 4.0 3.5 3.0 2.5 2.0 1.5 1.0 0.5
Chemical Shift (ppm)

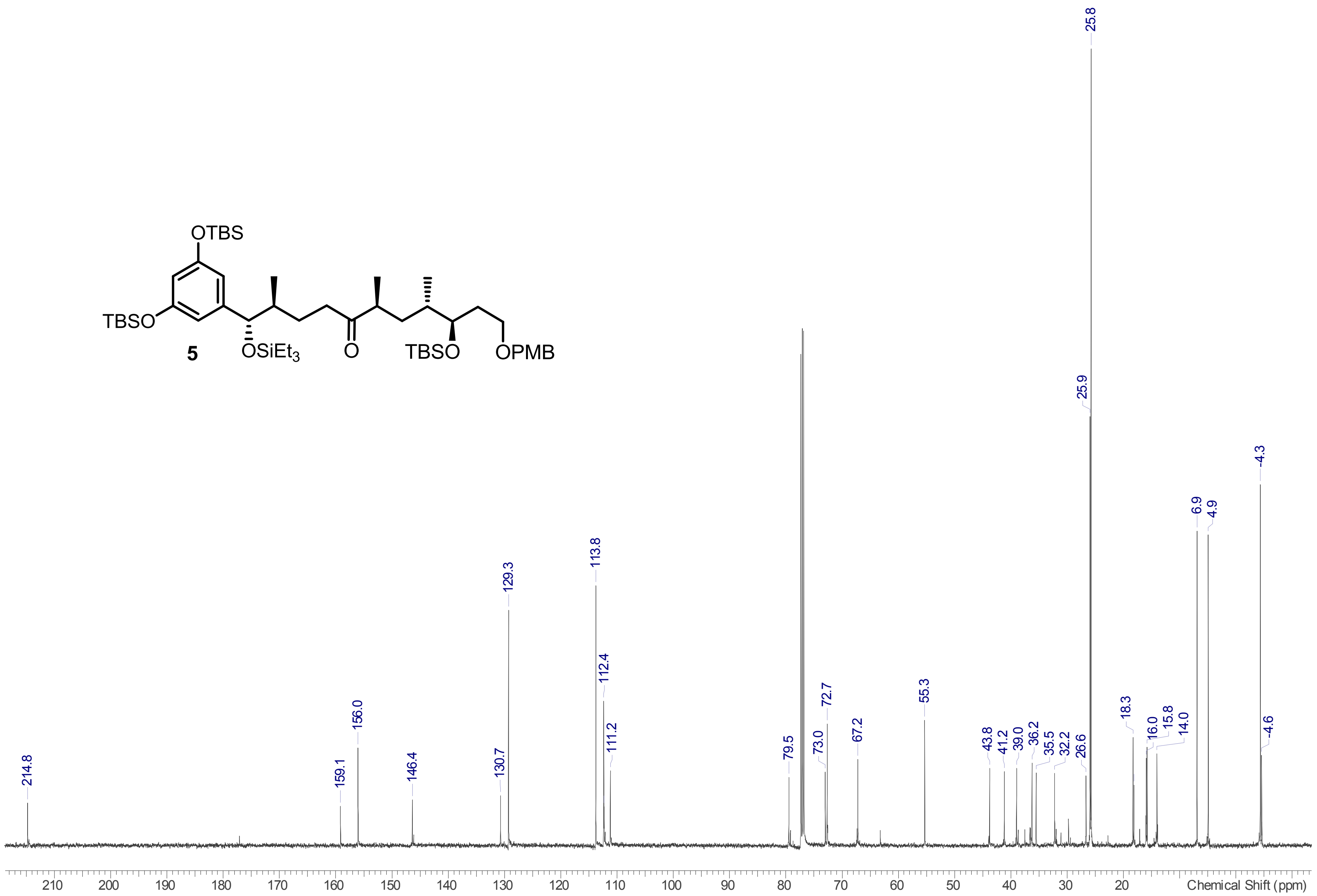
OTBS
TBSO
5
OSiEt3
O
TBSO
OPMB
214.8
159.1
156.0
146.4
130.7
129.3
113.8
112.4
111.2
79.5
73.0
72.7
67.2
55.3
43.8
41.2
39.0
36.2
35.5
32.2
26.6
25.9
25.8
18.3
16.0
15.8
14.0
6.9
4.9
-4.3
-4.6
210
200
190
180
170
160
150
140
130
120
110
100
90
80
70
60
50
40
30
20
Chemical Shift (ppm)

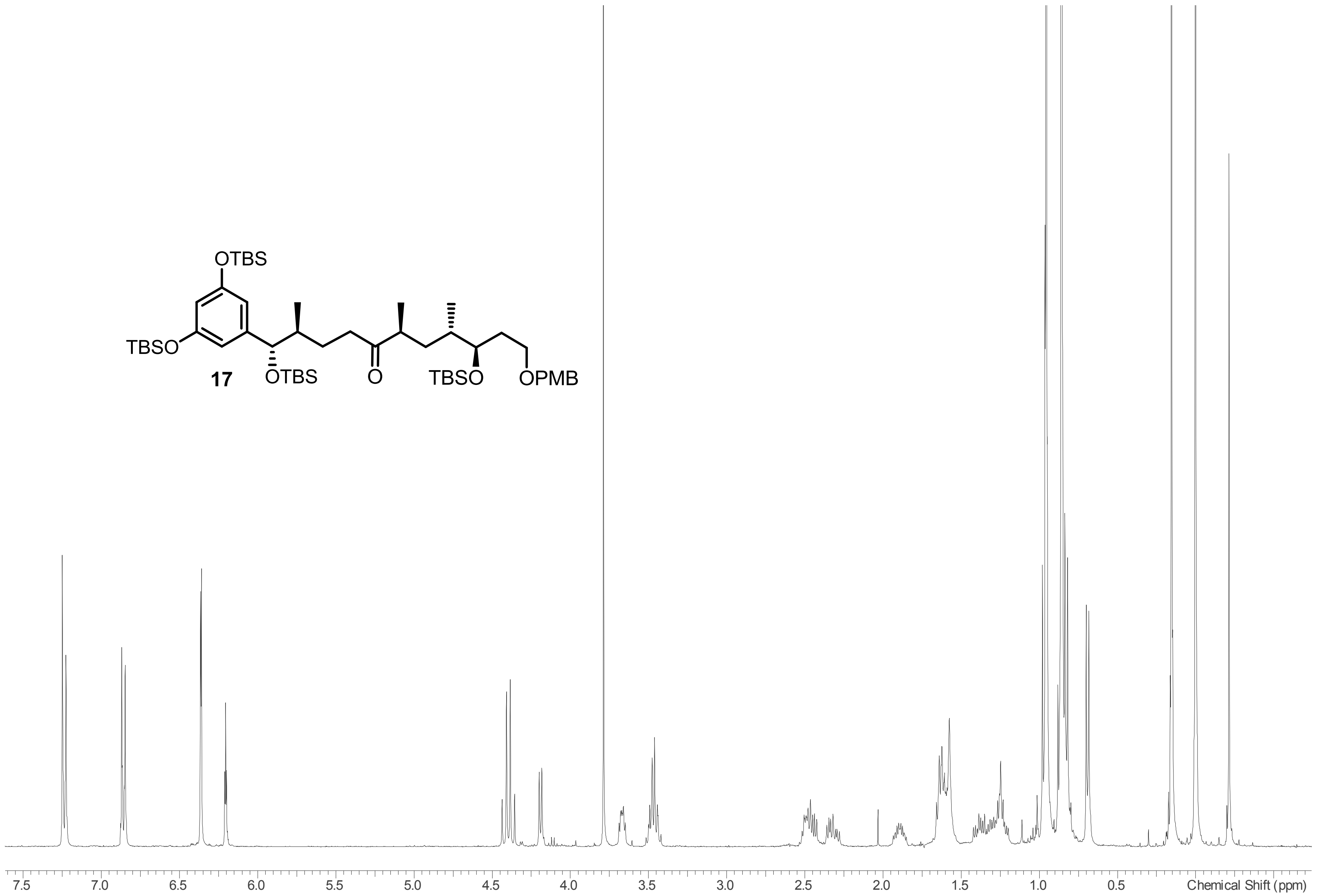
OTBS
TBSO
17
OTBS
O
TBSO
OPMB
7.5
7.0
6.5
6.0
5.5
5.0
4.5
4.0
3.5
3.0
2.5
2.0
1.5
1.0
0.5
Chemical Shift (ppm)

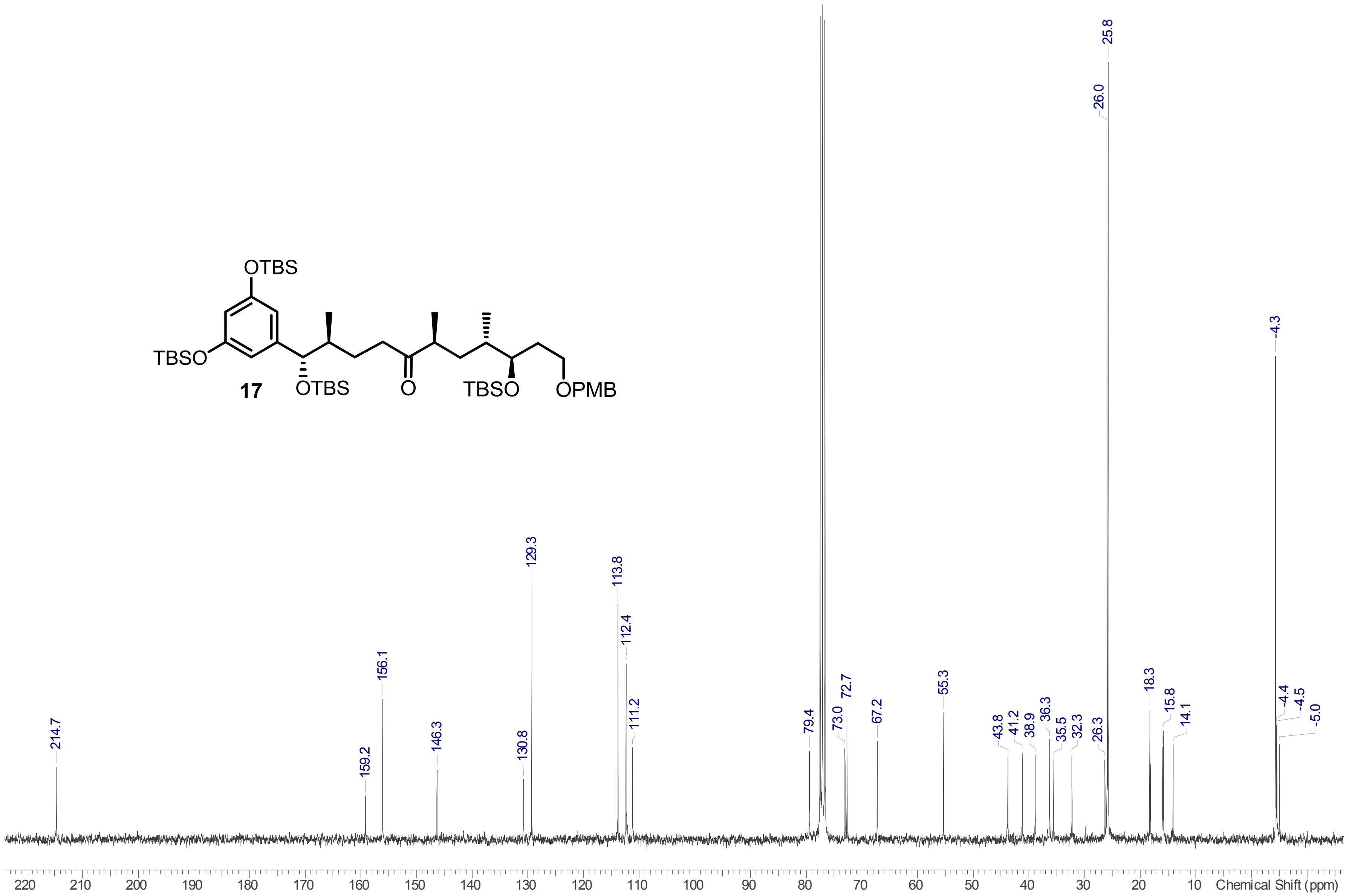
OTBS
TBSO
17
OTBS
O
TBSO
OPMB
214.7
159.2
156.1
146.3
130.8
129.3
113.8
112.4
111.2
79.4
73.0
72.7
67.2
55.3
43.8
41.2
38.9
36.3
35.5
32.3
26.3
26.0
25.8
18.3
15.8
14.1
-4.3
-4.4
-4.5
-5.0
220
210
200
190
180
170
160
150
140
130
120
110
100
90
80
70
60
50
40
30
20
10
Chemical Shift (ppm)

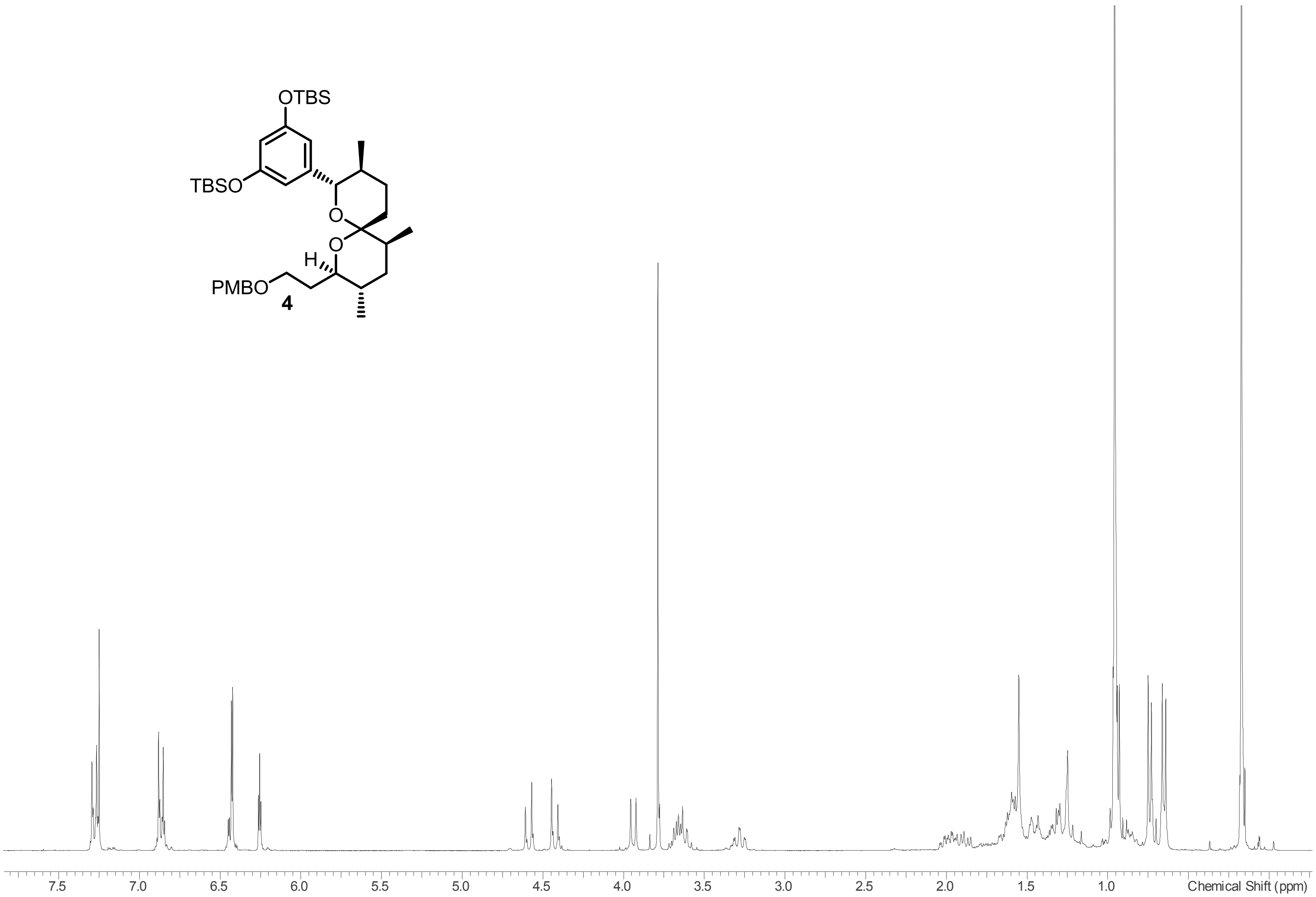

OTBS
TBSO
O
O
H
PMBO
4
7.5
7.0
6.5
6.0
5.5
5.0
4.5
4.0
3.5
3.0
2.5
2.0
1.5
1.0
Chemical Shift (ppm)

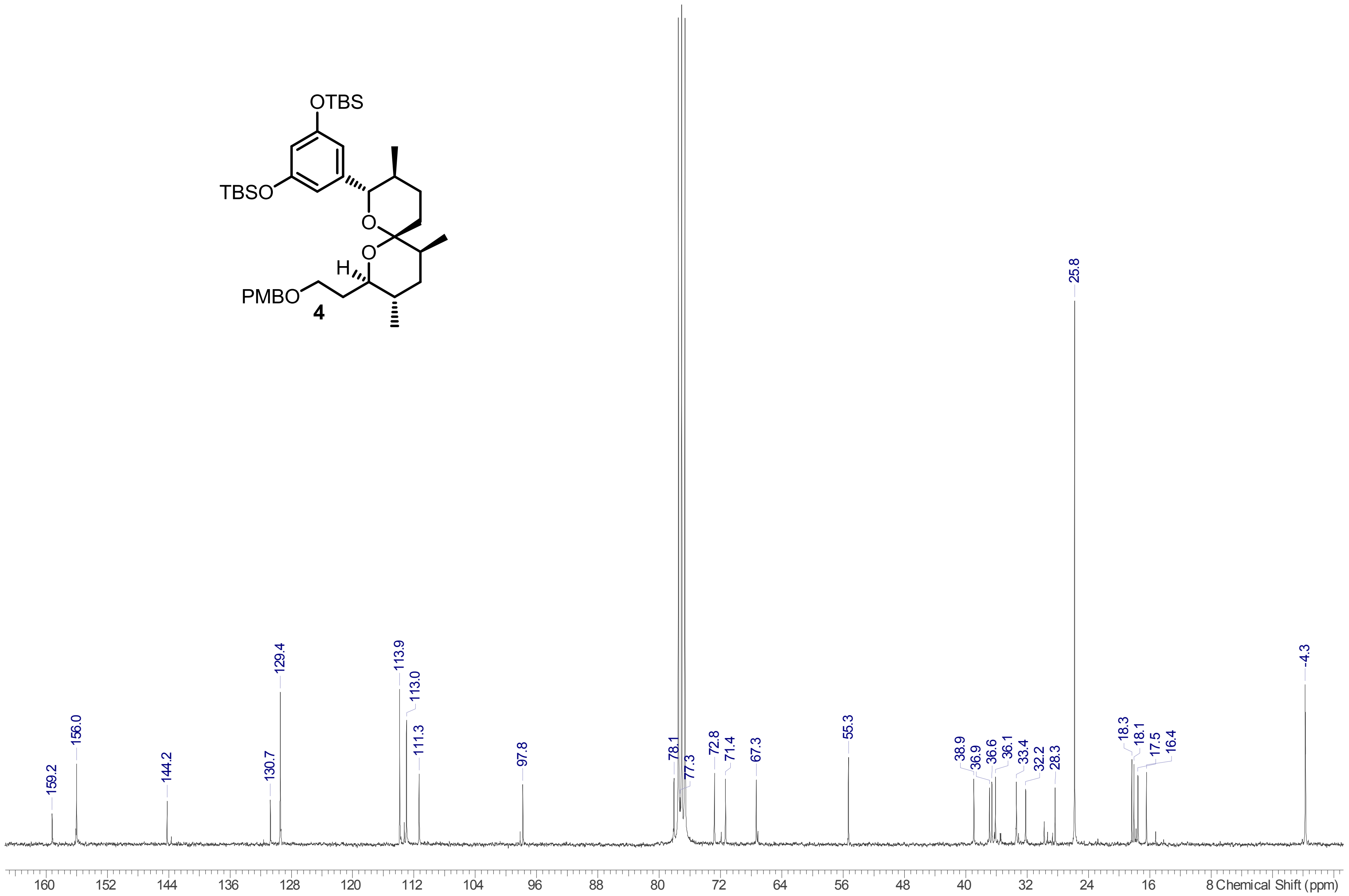
OTBS
TBSO
H
O
O
PMBO
159.2
156.0
144.2
130.7
129.4
113.9
113.0
111.3
97.8
78.1
77.3
72.8
71.4
67.3
55.3
38.9
36.9
36.6
36.1
33.4
32.2
28.3
25.8
18.3
18.1
17.5
16.4
-4.3
160
152
144
136
128
120
112
104
96
88
80
72
64
56
48
40
32
24
16
8 Chemical Shift (ppm)

4

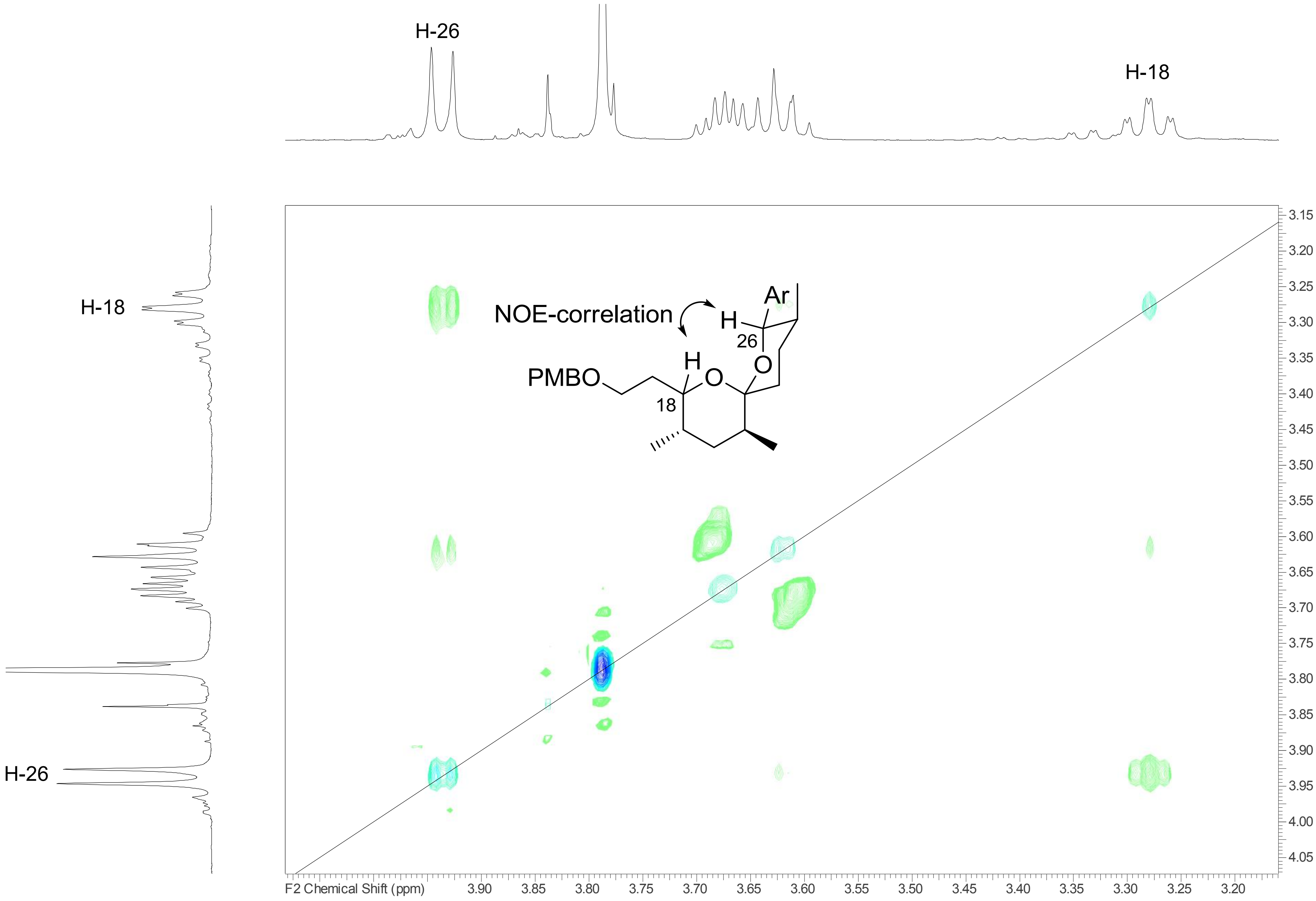
H-26
H-18
NOE-correlation
Ar
H
26
O
PMBO
H
O
18
F2 Chemical Shift (ppm)

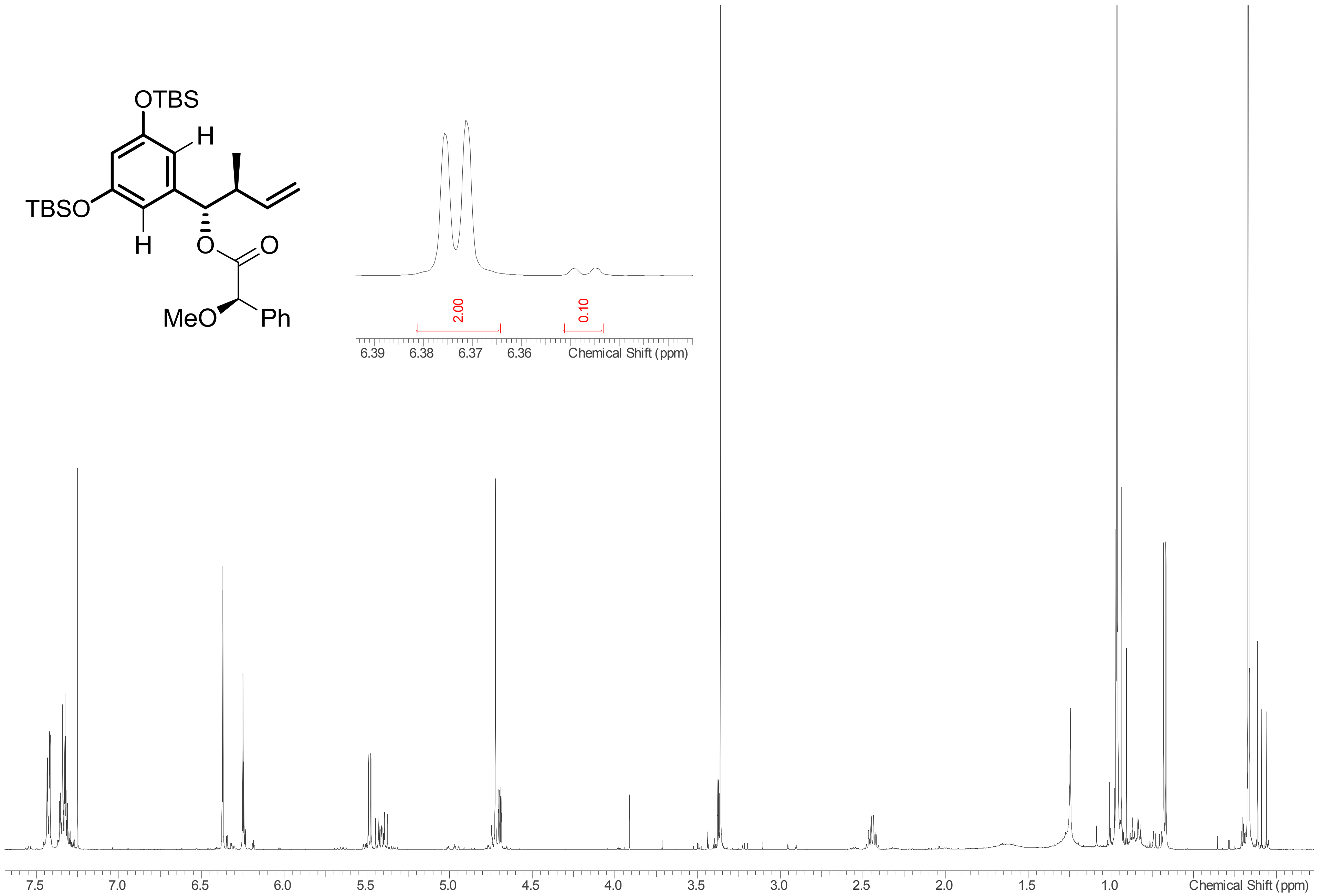
OTBS
H
TBSO
H
O
O
MeO
Ph
2.00
0.10
6.39
6.38
6.37
6.36
Chemical Shift (ppm)
7.5
7.0
6.5
6.0
5.5
5.0
4.5
4.0
3.5
3.0
2.5
2.0
1.5
1.0
Chemical Shift (ppm)
